# Migration deficits of the neural crest caused by CXADR triplication in a human Down syndrome stem cell model

**DOI:** 10.1038/s41419-022-05481-6

**Published:** 2022-12-05

**Authors:** Huanyao Liu, Shanshan Huang, Weijia Wang, Huiyan Wang, Weijun Huang, Zhichen Zhai, Ding Wang, Yubao Fan, Jiaqi Sun, Dairui Li, Weicheng Chiu, Xingqiang Lai, Jixiao Zeng, Qiong Ke, Tao Wang, Andy Peng Xiang, Yong Yuan, Xinchun Zhang, Weiqiang Li

**Affiliations:** 1 Center for Stem Cell Biology and Tissue Engineering, Key Laboratory for Stem Cells and Tissue Engineering, Ministry of Education, Sun Yat-Sen University, Guangzhou, Guangdong China; 2 Department of LabMedcine, Zhongshan people’s hospital, Zhongshan, Guangdong China; 3 Department of Obstetrics & Gynecology, Zhujiang Hospital, Southern Medical University, Guangzhou, China; 4 School of Materials Science and Engineering, South China University of Technology, Guangzhou, China; 5 Department of Obstetrics and Gynecology, Key Laboratory for Major Obstetric Diseases of Guangdong Province, The Third Affiliated Hospital of Guangzhou Medical University, Guangzhou, China; 6 Hospital of Stomatology, Guanghua School of Stomatology, Guangdong Provincial Key Laboratory of Stomatology, Sun Yat-sen University, Guangzhou, Guangdong China; 7 Department of Cardiology, The Eighth Affiliated Hospital, Sun Yat-sen University, Shenzhen, Guangdong China; 8 Department of Pediatric Surgery, Guangzhou Women and Children’s Medical Centre, Guangzhou, China; 9 Department of Cardiology, Zhongshan people’s hospital, Zhongshan, Guangdong China; 10 Guangdong Key Laboratory of Reproductive Medicine, Guangzhou, Guangdong China

**Keywords:** Stem cells, Diseases

## Abstract

Down syndrome (DS) is the most common chromosomal abnormality in live-born infants and is caused by trisomy of chromosome 21. Most individuals with DS display craniofacial dysmorphology, including reduced sizes of the skull, maxilla, and mandible. However, the underlying pathogenesis remains largely unknown. Since the craniofacial skeleton is mainly formed by the neural crest, whether neural crest developmental defects are involved in the craniofacial anomalies of individuals with DS needs to be investigated. Here, we successfully derived DS-specific human induced pluripotent stem cells (hiPSCs) using a Sendai virus vector. When DS-hiPSCs were induced to differentiate into the neural crest, we found that trisomy 21 (T21) did not influence cell proliferation or apoptosis. However, the migratory ability of differentiated cells was significantly compromised, thus resulting in a substantially lower number of postmigratory cranial neural crest stem cells (NCSCs) in the DS group than in the control group. We further discovered that the migration defects could be partially attributed to the triplication of the coxsackievirus and adenovirus receptor gene (CXADR; an adhesion protein) in the DS group cells, since knockdown of CXADR substantially recovered the cell migratory ability and generation of postmigratory NCSCs in the DS group. Thus, the migratory deficits of neural crest cells may be an underlying cause of craniofacial dysmorphology in individuals with DS, which may suggest potential targets for therapeutic intervention to ameliorate craniofacial or other neural crest-related anomalies in DS.

## Introduction

Down syndrome (DS), also known as trisomy 21 syndrome (T21), is the most common cause of congenital defects in newborn infants and is due to triplication of human chromosome 21 (HSA21) [1]. The phenotype of DS includes mental retardation, characteristic facies, congenital heart malformations, and gastrointestinal tract abnormalities [2]. Although the clinical features among individuals with DS vary in penetrance and expressivity, most people with DS present craniofacial deformities, which include decreased skull size, shortened mid-face, brachycephaly, and small maxilla and mandible. The resultant craniofacial malformation in people with T21 may significantly affect breathing, eating, and speaking [3]. Some DS individuals also have an increased incidence of congenital gut disorders such as Hirschsprung’s disease (HSCR), which is due to defects of the enteric nervous system [2]. The neural crest contributes to the majority of the bone, cartilage, connective tissue in the head, enteric nervous system (ENS), peripheral nervous system (PNS), sympathetic nervous system (SNS), and melanocytes throughout the body in vertebrates [4]. The above evidence indicates that neural crest defects may contribute to multiple aspects of the trisomic phenotypes in DS. Nonetheless, the underlying pathogenesis remains to be determined.

Primary human cells or tissues from individuals with DS are widely used to elucidate the underlying cellular and molecular mechanisms. The drawback is obvious, as the use of materials from individuals with trisomy of HSA21 is limited by the rarity of samples and experimental manipulability [5]. Several mouse models have also been developed for studies of the pathogenesis of DS. For example, Ts65Dn mice, the first and most popular segmental trisomy model, could partially replicate craniofacial abnormalities. However, Ts65Dn mice have 3 copies of 19 genes on Mmu17 that are not orthologous to HSA21. The Tc1 strain carries human chromosome 21 and has a number of DS-like phenotypes, including craniofacial dysmorphology. Nonetheless, many unsuspected deletions and rearrangements within the human chromosome in the TC1 strain have been identified. The above evidence suggests that the existing mouse models may not fully display the genetic and phenotypic characteristics of craniofacial deficits in human DS [6, 7]. In recent decades, reprogramming of human somatic cells to a pluripotent state has allowed the generation of human induced pluripotent stem cells (hiPSCs) [8], which share many characteristics with human embryonic stem cells (hESCs), including the ability to self-renew and differentiate into cells of all three germ layers. Therefore, hiPSC technology would be valuable in establishing disease models to study the mechanisms of disease pathogenesis.

DS-specific hiPSCs have been successfully established in numerous studies to reveal the underlying mechanisms of T21-relevant phenotypes. Tang et al. reported that the DSCAM/PAK1 pathway is involved in neurogenic deficits in DS using cerebral organoid models derived from DS-hiPSCs [9]. Nishinaka-Arai et al. found that DS-related transient abnormal myelopoiesis is attributed to a specific erythromegakaryocytic subpopulation with GATA1 mutation using T21 hiPSCs [10]. Nevertheless, none of these studies focused on the craniofacial manifestations and neural crest development in DS. In recent years, our group and others have developed highly efficient protocols for the derivation of cranial and vagal neural crest stem cells (NCSCs) from human pluripotent stem cells (hPSCs) for disease modeling and cell replacement therapy [11–13]. However, whether the in vitro differentiation of DS-hiPSCs could recapitulate the phenotype of craniofacial abnormalities and their related functional defects in NCSCs has not been determined. In this study, we successfully generated DS-specific hiPSCs from human amniotic fluid cells or embryonic fibroblasts with T21. The in vitro neural crest differentiation assay demonstrated migration defects and deficiency in the generation of migrating NCSCs in the T21 group compared to those of the euploid cell lines. Furthermore, RNA-sequencing analysis and functional assays indicated that triplication of the dosage-sensitive gene in HSA21, CXADR, was involved in the neural crest deficits that might be responsible for craniofacial deformation in individuals with DS.

## Results

### Generation and characterization of DS-specific hiPSCs

Three DS-hiPSC lines (DS1, DS2, and DS3) were successfully generated by reprogramming of embryonic fibroblasts (DS1) or amniotic fluid cells (DS2, DS3) using Sendai viral vectors. DS-hiPSCs had a high nucleus-to-cytoplasm ratio and prominent nucleoli and displayed a similar cell colony morphology and proliferative ability to hESCs during long-term in vitro culture (Supplementary Fig. 1a). Karyotyping analysis verified that all the DS-hiPSC lines showed trisomy for chromosome 21 (Supplementary Fig. 1b). In addition, the pluripotency of the DS-hiPSCs was verified by detection of pluripotency marker expression and differentiation assays. Immunofluorescence staining showed that the DS-hiPSCs expressed typical markers of pluripotent stem cells, including OCT4, NANOG, SSEA4, and TRA-1-81 (Supplementary Fig. 1c). When the DS-hiPSCs were induced to differentiate in vitro, the expression of the ectodermal marker tubulin b3 class III (TUBB3), mesodermal progenitor marker BRACHYURY (TBXT), and endodermal progenitor marker SOX17 could be detected in differentiated cells by immunostaining (Supplementary Fig. 1d). We next found that subcutaneous injection of the DS-hiPSCs into NOG mice could readily generate teratomas. Histological examination revealed that all three T21 hiPSC lines could generate cells and tissues of three germ layers in vivo, including ectoderm-derived neural tubes, mesoderm-derived cartilage, and endoderm-derived glandular epithelium (Supplementary Fig. 1e). The above evidence suggests that DS-hiPSCs possess the distinct characteristics of pluripotent stem cells, self-renewal, and pluripotency.

### T21 caused migration defects in the neural crest derived from DS-hiPSCs

**Fig. 1:**
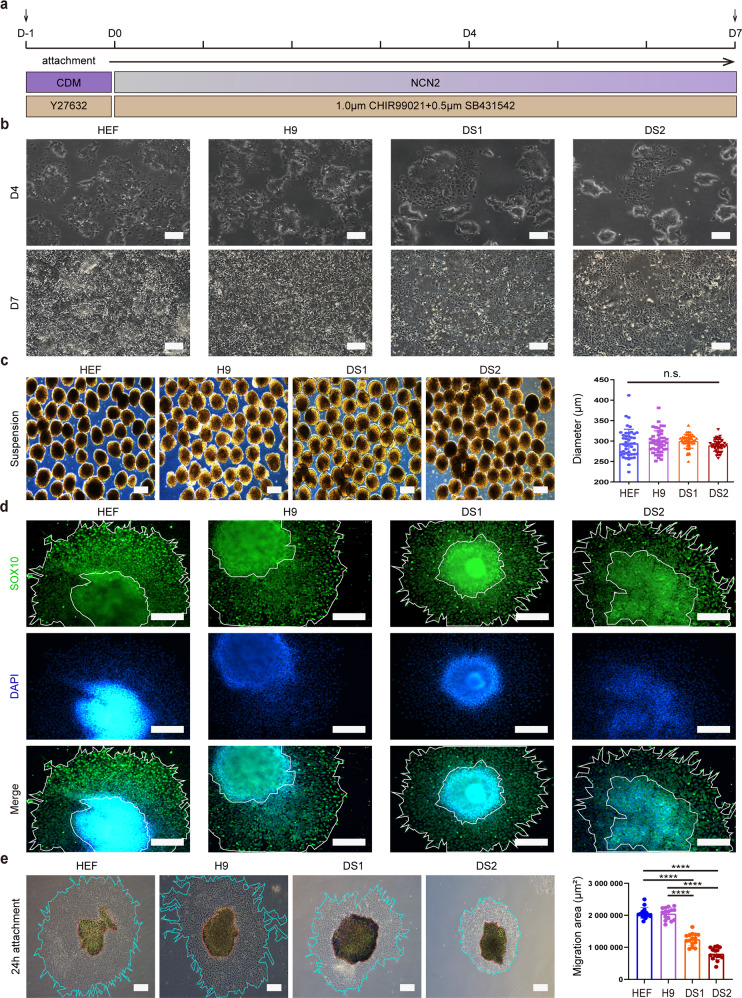
Migration defects of the neural crest derived from DS-hiPSCs. **a** Strategy for neural crest differentiation of hiPSCs in monolayer cultures. **b** The morphology of differentiated cells was observed using phase-contrast microscopy on day 4 (D4) and day 7 (D7) during neural crest differentiation. **c** Day 7 differentiated cells formed spheres when cultured in suspension for 24 h and the diameter of the spheres was calculated and compared (*n* = 45; one-way ANOVA; n.s. not significant). **d** The expression of a neural crest-specific marker (SOX10) was detected by immunostaining when spheres were seeded onto Matrigel-coated plates. **e** Cells were observed under a phase-contrast microscope at 24 h after attachment of spheres and cell migration area in different groups was calculated and compared (*n* = 15; two-tailed unpaired Student’s *t* test, ****P < 0.0001). Data are presented as the mean ± SD of three independent experiments. Scale bar: 250 μm.

hPSCs could be efficiently induced to differentiate into NCSCs by a well-defined differentiation protocol using CHIR99021- and SB431542-containing medium in monolayer cultures (Fig. 1a) [12, 14]. To explore whether DS-hiPSCs had neural crest differentiation defects, T21 cells were dissociated, replated onto Matrigel-coated plates at the same density and then cultured in differentiation medium for a 7-day protocol. H9 (hESCs), human embryonic fibroblast-derived hiPSCs (HEF-hiPSCs; HEF), and human amniotic fluid cell-derived hiPSCs (AFC-hiPSCs; AFC) [11] were used as control cell lines. Differentiated cells were observed under a phase contrast microscope, and we found that significantly more cells migrated out from the cell colonies in the control group than in the DS group on day 4 of differentiation (Fig. 1b). Moreover, these migrated cells displayed obviously different morphology from the undifferentiated hPSCs and presented a neural crest-like appearance of multipolar cells with narrow projections. Seven days later, multipolar cells in the control group proliferated or migrated quickly and became confluent, while the cells in the T21 group showed a reduced cell density (Fig. 1b).

**Fig. 2:**
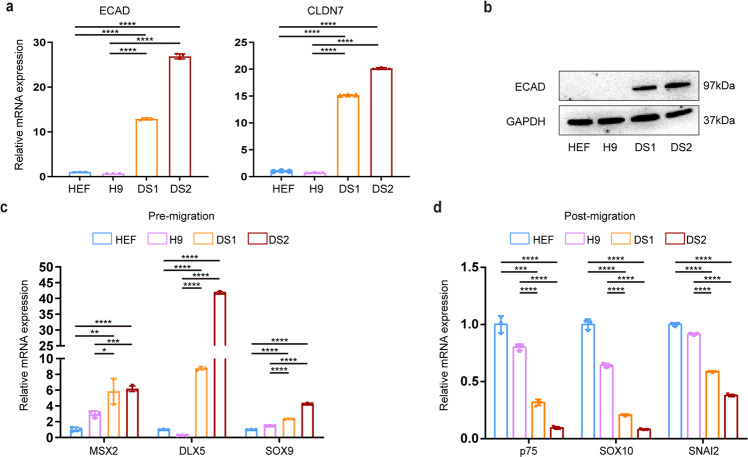
Migration- and EMT-related markers were differentially expressed between the trisomic and euploid cells. **a** qPCR assay for epithelial markers (ECAD, CLDN7) in day 7 cells. **b** Western blotting analysis for the protein expression level of ECAD in day 7 cells. **c** qPCR assay for premigratory markers (MSX2, DLX5, SOX9) in day 7 cells. **d** qPCR assay for postmigratory markers (p75, SOX10, SNAI2) in day 7 cells. Data are presented as the mean ± SD of three independent experiments. *n* = 3. *P < 0.05, **P < 0.01, ***P < 0.001, ****P < 0.0001, two-tailed unpaired Student’s *t* test.

We first determined whether cells in the DS group have a reduced proliferative ability or increased apoptosis during neural crest specification. Anti-Ki67 immunostaining showed that the number of Ki67^+^ cells in the DS group was similar to that in the control group on day 7 (Supplementary Fig. 2a, b). The Cell Counting Kit-8 (CCK-8) assay also revealed no significant difference in cell proliferation between the control and DS groups during NCSCs differentiation (Supplementary Fig. 2c). In addition, both immunostaining for active caspase 3 and terminal deoxynucleotidyl transferase (TdT) dUTP nick-end labeling (TUNEL) assays indicated that the apoptosis rate on day 7 was comparable between the trisomic and euploid cells (Supplementary Fig. 2d, e). To further determine whether T21 affects the migratory ability of differentiated cells of the DS group, we collected day 7 cells and cultured them in suspension to form spheres for 1 day. We found that the numbers and diameters of the spheres were similar between the control and DS groups (Fig. 1c; Supplementary Fig. 3a). Then, the spheres were seeded on Matrigel-coated plates in neural crest culture medium (NCCM) and cultured for 24 h. Cells rapidly migrated out from the spheres and highly expressed the neural crest-specific marker SOX10, and we found that the cell migration area in the DS group was drastically lower than that in the control group (Fig. 1d, e; Supplementary Fig. 3b, c). Previous studies reported that premigratory neural crest cells undergo epithelial-to-mesenchymal transition (EMT) and become migrating NC cells, which highly express migration-related genes [15]. We, therefore, detected the expression of EMT-related genes in day 7 cells. We found that the expression of epithelial markers, including E-cadherin (ECAD) and Claudin 7 (CLDN7) (Fig. 2a, b; Supplementary Fig. 3d), and premigratory markers (MSX2, DLX5, SOX9; Fig. 2c; Supplementary Fig. 3d) was strongly upregulated, while the transcript levels of postmigratory markers (p75, SOX10, SNAI2) were notably decreased in the DS group compared to the control group (Fig. 2d; Supplementary Fig. 3h).

Previous studies have also demonstrated that hiPSCs and hESCs can differentiate into the neural crest through the formation of embryoid bodies (EBs) (Supplementary Fig. 4a) [11]. We then investigated whether the EB-based protocol resulted in a similar phenotype of defective migration in trisomic cells as the monolayer method. hPSCs were suspended in AggreWell plates to form uniform-sized EBs (Supplementary Fig. 4b). No distinct differences in the quantity of EBs formed by different cell lines were observed. Nonetheless, the average EB size formed by the trisomic cells was substantially smaller than that of the control group (Supplementary Fig. 4c, d), which was similar to the results of a previous report and due to abnormal neurogenesis in cells in the DS group [9]. Then, the EBs were attached to Matrigel-coated culture dishes in neural crest culture medium (NCCM) and cultured for 48 h, and the cell migration area between 24 and 48 h was measured and compared. We found that EBs could attach readily and that cells in all groups could subsequently migrate out of the EBs 24 h after seeding in all groups (Supplementary Fig. 4e). However, the migration area in the DS group between 24 and 48 h was significantly smaller than that of the control group (Supplementary Fig. 4e–g). Time-lapse imaging also revealed a substantially reduced migration distance and speed in individual cells in the DS group compared to the control cells (Supplementary Fig. 4h–i). Taken together, the above evidence reveals an impaired migratory ability in T21 cells during neural crest development.

### Generation of the postmigratory neural crest was severely impaired in DS-hiPSCs

**Fig. 3:**
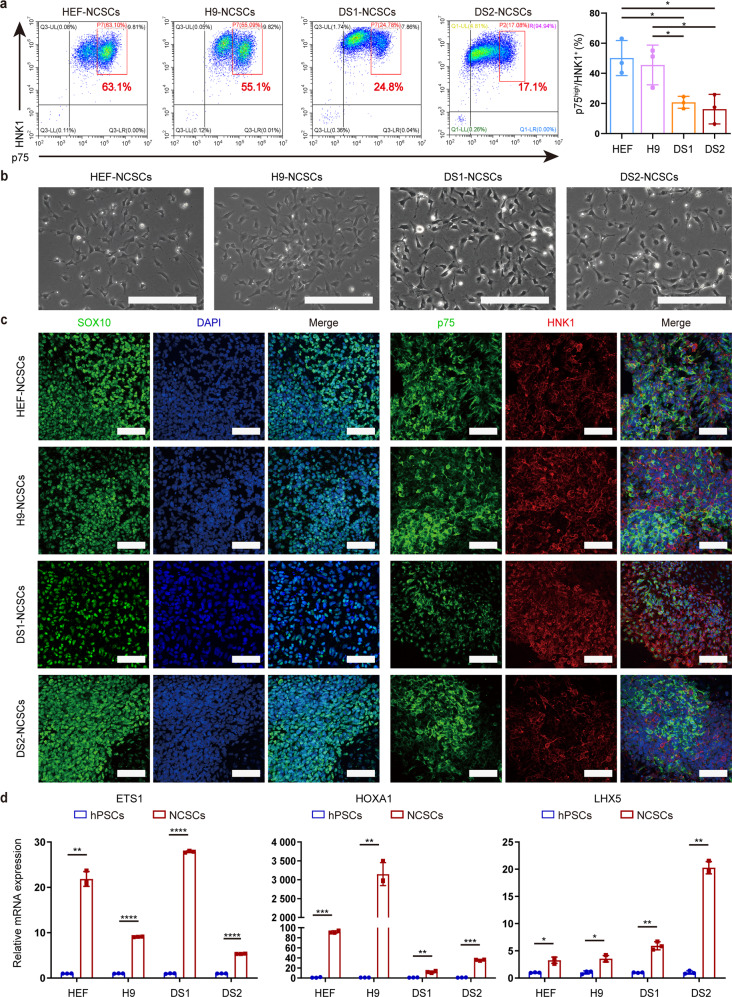
Generation of migrating NCSCs was inhibited in the DS group. **a** The percentage of postmigratory NCSCs (p75^high^/HNK1^+^) generated by monolayer protocol was detected by FACS. **b** FACS-enriched NCSCs maintained typical neural crest morphology during in vitro culture. Scale bar: 250 μm. **c** The expression of the neural crest-specific markers (SOX10, p75, HNK1) in isolated NCSCs was detected by immunostaining. Scale bar: 100 μm. **d** The expression of cranial neural crest-specific markers (ETS1, HOXA1, and LHX5) in NCSCs was detected by qPCR. Data are presented as the mean ± SD of three independent experiments. *n* = 3. *P < 0.05, **P < 0.01, ***P < 0.001, ****P < 0.0001, two-tailed unpaired Student’s *t* test.

The migration defects in trisomic cells prompted us to investigate whether T21 inhibits the generation of postmigratory/migrating neural crest from DS-hiPSCs. Previous studies have demonstrated that NCSCs (including premigratory and postmigratory NCSCs) coexpressed the cell surface markers p75 and HNK1, while postmigratory NCSCs were defined as the p75^high^/HNK1^+^ population when analyzed by fluorescence-activated cell sorting (FACS) [12, 16, 17]. We then performed FACS analysis of the differentiated cells during neural crest commitment in a monolayer system on day 7. The results showed that although the differentiation efficiency for the total neural crest population (p75^+^/HNK1^+^ cells) was similar in the DS and control groups, DS-hiPSCs exhibited impaired differentiation of the migrating neural crest, as the percentage of HNK1^+^/p75^high^ in the T21 group was significantly lower than that in the euploid group (HEF: 50.1 ± 11.7%; H9: 45.6 ± 13.2%; AFC: 50.1 ± 2.2%; DS1: 20.8 ± 4.0%; DS2: 16.2 ± 9.8%; DS3: 7.4 ± 0.8%) (Fig. 3a; Supplementary Fig. 5a). Subsequently, the DS-NCSCs were enriched by FACS and propagated in adherent monoculture using NCCM. We found that the DS-NCSCs displayed the typical cellular morphology of the neural crest during in vitro culture (Fig. 3b; Supplementary Fig. 5b), and most of these cells showed expression of neural crest-specific markers, including SOX10, p75, and HNK1, as illustrated by immunofluorescence staining (Fig. 3c; Supplementary Fig. 5c). qPCR analysis revealed significantly upregulation of the expression of cranial neural crest markers (ETS1, HOXA1, and LHX5) in NCSCs compared to undifferentiated hPSCs (Fig. 3d; Supplementary Fig. 5d). Using the EB-based neural crest differentiation protocol, we also observed the compromised developmental potential of postmigratory NCSCs from DS-hiPSCs (HEF: 14.4 ± 0.9%; H9: 21.3 ± 5.4%; DS1: 1.1 ± 0.4%; DS2: 0.7 ± 0.4%; Supplementary Fig. 6a). Nonetheless, the DS-NCSCs enriched from EBs showed similar cell morphology and marker expression patterns as the control NCSCs (Supplementary Fig. 6b, c). These results indicate that T21 leads to a decreased number of postmigratory NCSCs due to defects in migration, as described above.

### Multilineage differentiation of DS-NCSCs

Individuals with DS were also found to exhibit dysfunction of the PNS, SNS, and ENS, which may suggest a reduced number or diminished differentiation capacity of the neural crest cells in DS [18, 19]. We next investigated whether T21 influences the multilineage differentiation potential of the DS-NCSCs. For neuronal differentiation, cells were cultured in induction medium containing neurotropic factors, including BDNF, GDNF, and NGF. After 3–4 weeks, the NCSCs gradually underwent morphological changes and adopted a neuron-like appearance with multipolarity and long processes. Immunostaining assays confirmed that over 50% of the differentiated cells in either the DS or control group coexpressed peripheral neuronal-specific markers such as peripherin (PRPH) and TUBB3 (TUJ1) (Supplementary Fig. 7a). NCSCs were also induced to differentiate into Schwann cells by exposure to medium containing CNTF, neuregulin, and db-cAMP for 2–3 weeks. Cells with the typical bipolar spindle-shaped morphology of Schwann cells subsequently emerged. Immunofluorescence assays revealed that most of the differentiated cells in both the trisomic and euploid groups coexpressed the Schwann cell markers GFAP and S100B (Supplementary Fig. 7b). These results indicate that T21 might not affect the peripheral neuronal or glial commitment of NCSCs.

The neural crest was reported to contribute to the facial skeleton in vertebrates and could be directed toward mesenchymal lineages [20, 21]. Here, we tested whether the DS-NCSCs had mesenchymal differentiation potential similar to that of the control group. NCSCs were cultured in MesenCult^TM^-ACF Plus Medium for 3–4 weeks. The cell morphology changed significantly, and mesenchymal-like cells emerged with parallel or spiral arrangement (Supplementary Fig. 8a). FACS analysis for cell surface marker expression showed that the differentiated cells from both the DS and control groups highly expressed markers typical of mesenchymal stem cells (MSCs), including CD29, CD44, CD73, CD105, and CD166, but less than 2% of the cells were positive for CD34 or CD45 (Supplementary Fig. 8b). Moreover, we found that the MSC-like cells in both the DS and control groups could be efficiently induced to differentiate into osteoblasts and adipocytes, as illustrated by Alizarin Red S staining and Oil Red O staining, respectively (Supplementary Fig. 8c). The above preliminary data suggest that trisomic cells possess similar differentiation properties toward MSCs, osteoblasts, and adipocytes. Interestingly, the DS-NCSCs showed reduced cartilaginous matrix formation compared to the control NCSCs during chondrogenic commitment, as detected by toluidine blue staining, indicating the compromised chondrogenic differentiation potential in the DS group (Supplementary Fig. 8c). This phenomenon may be associated with DS phenotypes such as hypoplasia of cartilage-derived basilar, facial, and nasal bones [22], but the underlying molecular mechanism remains to be elucidated.

### Differential gene expression patterns between DS-NCSCs and control-NCSCs

**Fig. 4:**
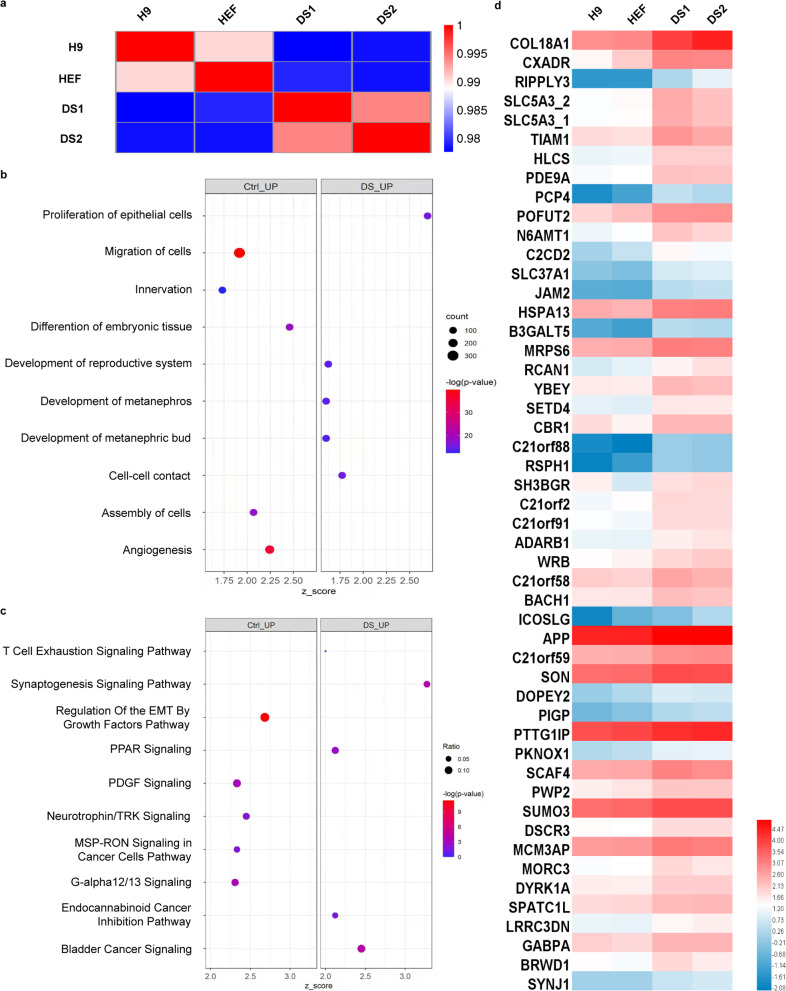
Genome-wide transcriptional profile analysis of DS-NCSCs. **a** Pearson’s correlation coefficients of pairwise comparisons were calculated for all expressed genes in NCSCs derived from the control group and the DS group. **b** IPA functional annotation of all differentially expressed mRNAs. The dot plot of partially enriched functions. The color intensity of the nodes indicates the degree of IPA function enrichment. The horizontal axis indicates the gene ratio as the proportion of differentially expressed genes in the whole gene set. The size represents the number counts in a certain function. **c** IPA canonical pathway analysis of all differentially expressed mRNAs. The dot plot of partially enriched pathways. The color intensity of the nodes indicates the degree of canonical IPA pathway enrichment. The horizontal axis indicates the gene ratio as the proportion of differentially expressed genes in the whole gene set. The size represents the number counts in a certain pathway. **d** The top 50 genes with upregulated expression on HSA21 in the DS group compared to the control group.

To further determine the possible mechanism underlying the migration defects and characterize the properties of trisomic NCSCs in detail, we performed a genome-wide transcriptional profile analysis of NCSCs in both groups (GSE190305 for DS1-NCSCs and DS2-NCSCs; GSE132857 for H9-NCSCs and HEF-NCSCs). To investigate the global relatedness of the gene expression profiles of NCSCs between the control and DS groups, we calculated the coefficients of determination (R2) for all expressed genes. As expected, highly similar gene expression profiles were detected between samples from the control group and the DS group, indicating the excellent reproducibility of our neural crest differentiation protocol. Nevertheless, relatively lower similarity in the transcriptome was noted between the control and DS cells (Fig. 4a). We first identified all differentially expressed genes (DEGs; with RPKM value more than 1). About 460 DEGs were identified between control group and DS group, and most of these genes are not located on HSA21 (Control-high genes, Supplementary Table 7; DS-high genes, Supplementary Table 8). In addition, IPA analysis (IPA Functions Annotation) of differentially expressed genes showed that approximately 200 migration-related genes were significantly enriched in the control cells rather than the DS cells (Fig. 4b). Furthermore, IPA canonical pathway analysis indicated that the “regulation of EMT by growth factor pathway” was much stronger in euploid cells than in trisomic cells (Fig. 4c). Since migration defects in DS were detected in vitro, we used Ingenuity Pathway Analysis (IPA) software to analyze the expression of genes associated with “cell movement” and “adhesion”. The top 20 genes with upregulated expression and the top 20 genes with downregulated expression related to “cell movement” or ‘adhesion’ were identified (Supplementary Fig. 9a, b). More importantly, to determine the genes responsible for the migration defects in the neural crest in the DS group, we analyzed the top 50 genes on HSA21 with upregulated expression in the DS group (Fig. 4d). The results showed that these genes included COL18A1, CXADR, and others, which were increased more than 2-fold in the DS cells compared to the control cells. These transcriptome analyses support the notion that the migration defects of the DS-NCSCs might be mediated by the upregulation of gene expression on HSA21.

### CXADR overexpression is involved in the migration defects of DS-NCSCs

To further explore the genes responsible for the impairment of migration during neural crest development in T21, we selected three genes on chromosome 21, CXADR, COL18A1, and SUMO3, to investigate the relationship between their gene expression levels and the migration deficit in DS-NCSCs, which were thought to be associated with migration or neural crest specification. For instance, CXADR, the coxsackievirus and adenovirus receptor gene, is a cell adhesion molecule and tight junction protein and has been reported to regulate epithelial–mesenchymal plasticity in breast cancer cells [23]. Similarly, previous work showed that small ubiquitin-like modifier 3 (SUMO3) could catalyze the SUMOylation of a RhoGAP protein, ARHGAP21, which is known to be involved in cell migration [24]. For COL18A1, several reports demonstrated that this gene was related to the structural stability of basement membranes and the inhibition of endothelial migration through its C-terminal fragment, endostatin [25]. Nonetheless, it remains unclear whether triplication of CXADR, SUMO3, or COL18A1 contributes to neural crest deficits in DS.

**Fig. 5:**
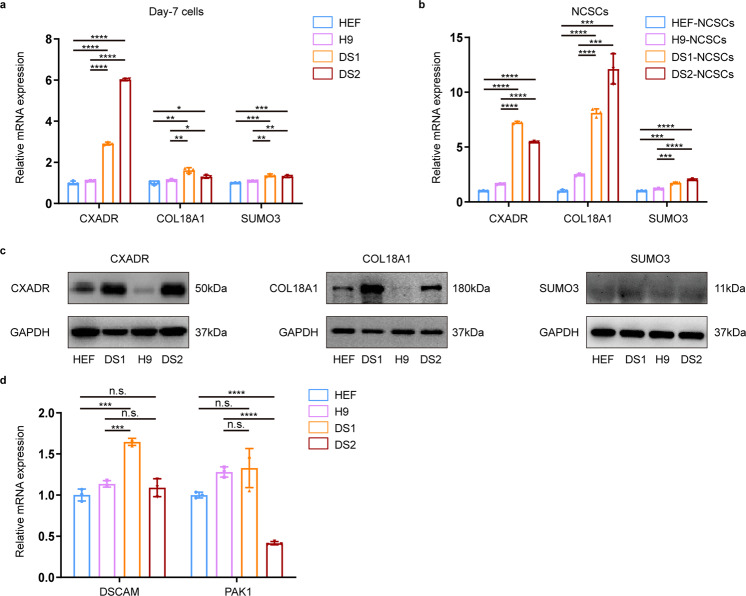
The expression of CXADR, COL18A1, and SUMO3 was upregulated in DS-NCSCs compared to control NCSCs. **a** qPCR assay for CXADR, COL18A1, and SUMO3 expression in day 7 cells before FACS enrichment. **b** qPCR assay for CXADR, COL18A1, and SUMO3 expression in p75^high^/HNK1^+^ NCSCs enriched by FACS. **c** The protein level of CXADR, COL18A1, and SUMO3 in DS and control cells was detected by western blotting. **d** qPCR assay for the mRNA expression of DSCAM and PAK1. Data are presented as the mean ± SD of three independent experiments. *n* = 3. *P < 0.05, **P < 0.01, ***P < 0.001, ****P < 0.0001, two-tailed unpaired Student’s *t* test.

**Fig. 6:**
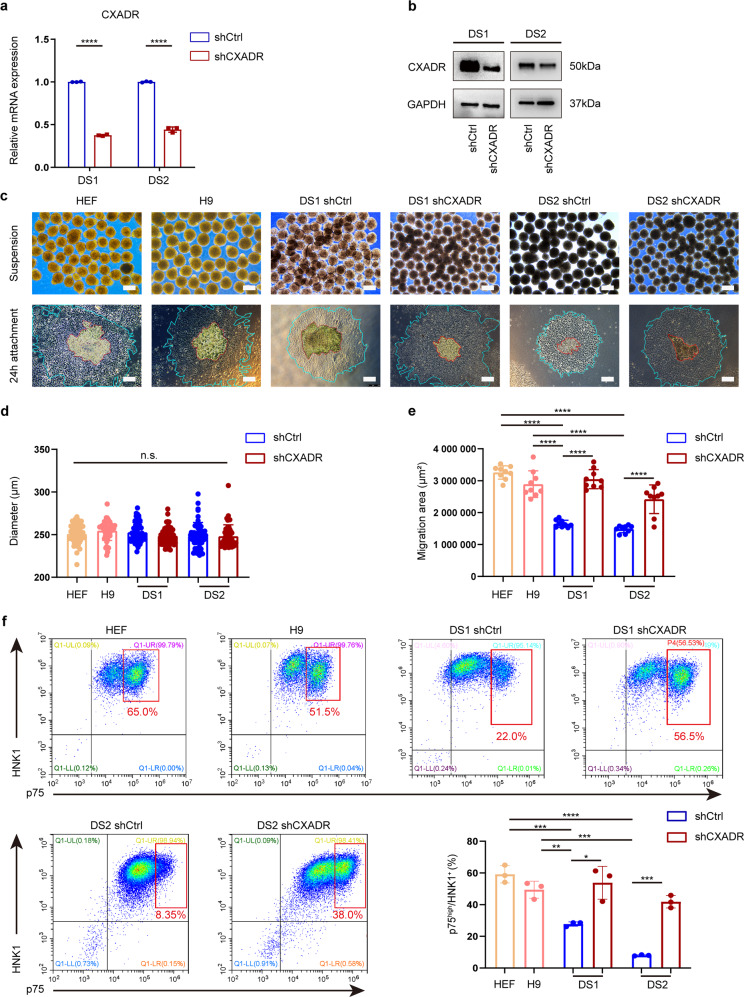
Migration defects in trisomic NCSCs were ameliorated after knockdown of CXADR. **a** qPCR assay for CXADR expression between non-target DS control (DS shCtrl) and CXADR knockdown DS group (DS shCXADR). **b** The efficiency of the shRNA-mediated downregulation of CXADR expression in DS-hiPSCs was assessed at the protein level. **c** Spheres were formed by day 7 differentiated cells derived from different groups (euploid group, DS shCtrl group, and DS shCXADR group) and then grew in attachment culture for 24 h. Scale bar: 250 μm. **d** The diameter of the spheres formed by day 7 cells were calculated and compared. *n* = 50. **e** The cell migration area was quantified and compared between different groups. *n* = 12. **f** The percentage of postmigratory NCSCs (p75^high^/HNK1^+^) derived from different groups was measured by FACS. *n* = 3. Data are presented as the mean ± SD of three independent experiments. *P < 0.05, **P < 0.01, ***P < 0.001, ****P < 0.0001, one-way ANOVA (**d**) and two-tailed unpaired Student’s *t* test (**a**, **e**, **f**).

Quantitative real-time PCR (qRT-PCR) analyses of day 7 differentiated cells and FACS-enriched NCSCs further confirmed that the mRNA expression of CXADR and COL18A1 was indeed significantly upregulated in the DS group compared to that of the euploid NCSCs (Fig. 5a, b; Supplementary Fig. 10a, b). Western blotting results also indicated that the protein levels of CXADR and COL18A1 were substantially increased in T21 cells (Fig. 5c; Supplementary Fig. 10c). However, the expression levels of SUMO3 were mildly increased in T21 cells compared to euploid cells (Fig. 5a–c; Supplementary Fig. 10a–c). Moreover, we found that the expression of DSCAM and PAK1, which have been reported to be associated with migration deficits of GABAergic neurons in individuals with DS [26], was not consistently expressed in DS cell lines (Fig. 5d). These results suggest that DSCAM and PAK1 are not involved in neural crest defects in DS. We subsequently knocked down the expression of CXADR, COL18A1 or SUMO3 in DS-hiPSCs by shRNA lentivirus transduction. qRT-PCR and western blotting demonstrated that the mRNA and protein levels of these genes were successfully downregulated after RNA interference (Fig. 6a, b; Supplementary Fig. 11a–d; Supplementary Fig. 12a). These cell lines were then induced to differentiate into the neural crest lineage by a monolayer protocol, and the percentage of migrating NCSCs (p75^high^HNK1^+^ cells) was measured by FACS. The results showed that knockdown of COL18A1 or SUMO3 did not significantly improve the derivation of migrating NCSCs from DS-hiPSCs (DS1 shCOL18A1: 19.2 ± 0.1%, DS1 shSUMO3: 13.3 ± 6.9%, DS1 shCtrl: 25.6 ± 1.9%; DS2 shCOL18A1: 17.7 ± 4.5%, DS2 shSUMO3: 10.9 ± 1.7%, DS2 shCtrl: 10.9 ± 0.3%) (Supplementary Fig. 11e, f). These results indicate that overexpression of SUMO3 and COL18A1 may not be associated with decreased migration of DS-NCSCs.

**Fig. 7:**
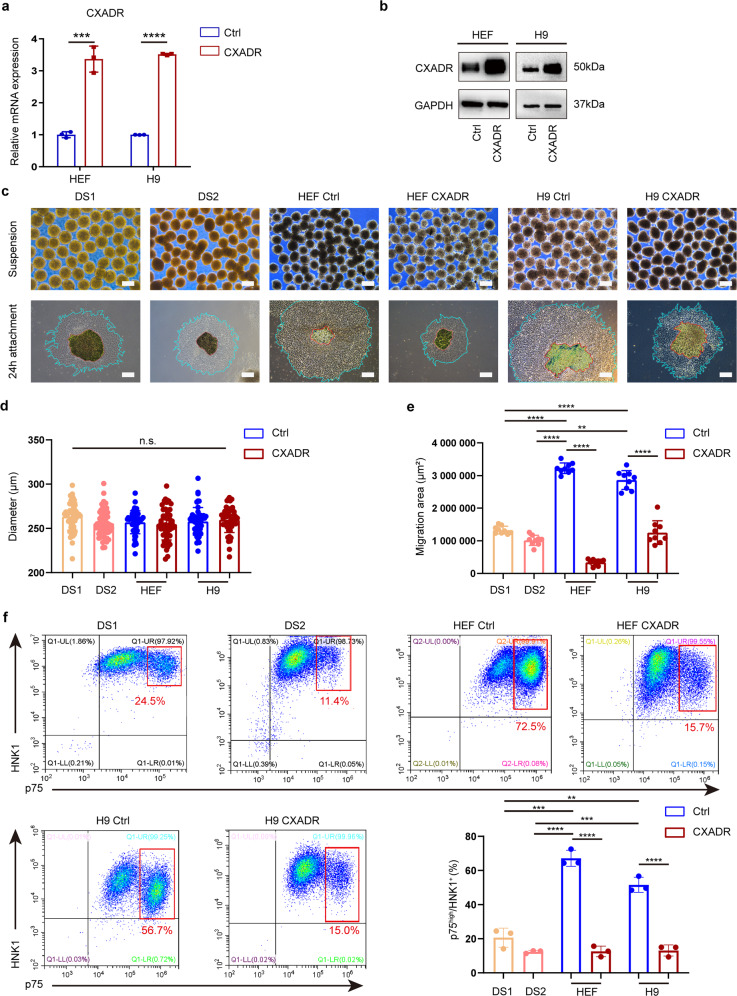
Overexpression of CXADR impaired the migratory capacity of control NCSCs. **a** qPCR assay for CXADR expression in the control hPSCs after lentiviral transduction. **b** Protein expression of CXADR was evaluated after lentiviral transduction. **c** Spheres were formed by day 7 differentiated cells from different groups (DS group, Ctrl euploid group, and CXADR-overexpression euploid group) and then grew in attachment culture for 24 h. Scale bar: 250 μm. **d** The diameter of the spheres formed by day 7 differentiated cells were calculated and compared between different groups. *n* = 50. **e** The cell migration area was quantified and compared between different groups. *n* = 12. **f** The percentage of postmigratory NCSCs (p75^high^/HNK1^+^) derived from between different groups was detected by FACS. *n* = 3. Data are presented as the mean ± SD of three independent experiments. *P < 0.05, **P < 0.01, ***P < 0.001, ****P < 0.0001, one-way ANOVA (**d**) and two-tailed unpaired Student’s *t* test (**a**, **e**, **f**).

Intriguingly, when day 7 cells were cultured in suspension and then replated onto a Matrigel-coated surface, we found that the knockdown of CXADR did not affect the formation or diameters of spheres (Fig. 6c, d; Supplementary Fig. 12b). Interestingly, the cell migration ability of the DS shCXADR group was substantially restored compared with that of the DS shCtrl group, and similar migration area was detected between DS shCXADR group and euploid control group (Fig. 6c, e; Supplementary Fig. 12c). Moreover, we discovered that comparable cell numbers of postmigratory NCSCs were generated in DS shCXADR group and euploid control group, which was both significantly higher (2- to 3-fold) than that in DS shCtrl group (HEF: 59.2 ± 5.5%, H9: 49.4 ± 5.4%, AFC: 39.0 ± 2.3%; DS1 shCXADR: 52.9 ± 6.5%, DS2 shCXADR: 41.8 ± 4.0%, DS3 shCXADR: 32.3 ± 0.9%; DS1 shCtrl: 20.6 ± 3.8%, DS2 shCtrl: 8.0 ± 0.4%, DS3 shCtrl: 6.0 ± 1.0%) (Fig. 6f; Supplementary Fig. 12d). To confirm this observation, we also overexpressed CXADR in H9, HEF-hiPSCs and AFC-hiPSCs and investigated whether forced expression of CXADR in euploid cells would cause a similar phenotype of migration defects observed in the DS group. A lentiviral vector for overexpression of CXADR was therefore constructed and used to transduce the control cell lines HEF, H9, and AFC. Quantitative PCR (qPCR) detection and western blotting both revealed that CXADR was successfully overexpressed in these cells (Fig. 7a, b; Supplementary Fig. 12e). Further studies revealed that CXADR overexpression led to greatly impaired migratory capacity of control NCSCs and reduced numbers of migrating NCSCs from H9 and HEF-hiPSCs, a phenomenon consistent with that in DS group (Fig. 7c–f; Supplementary Fig. 12f–h).

In addition, to examine the potential molecular mechanism of how CXADR regulates cell migration, we performed RNA-Seq of shCtrl and shCXADR DS-NCSCs (HRA003303). The results manifested that CXADR knockdown could markedly rescue the migration activity of DS-NCSCs, as shown by the enhanced function of “invasion of tissue” and “invasion of cells” in DS shCXADR cells than DS shCtrl cells (Supplementary Fig. 13a). IPA canonical pathway analysis revealed that PDGF Signaling, NFκB Signaling, and others were noticeably strengthened, while WNT/β-catenin Signaling, PI3K/AKT Signaling, and others were compromised in DS shCXADR group compared with DS shCtrl group (Supplementary Fig. 13b). Nonetheless, whether these pathways are involved in the migration defects in DS-NCSCs needs further elucidation.

Meanwhile, to further validate the above phenomenon, we performed CXADR gene knockout (KO) in DS cell lines using CRISPR/Cas9 gene editing tool. qPCR and western blotting verified that the expression of CXADR was strongly inhibited in all CXADR KO cell lines (Supplementary Fig. 14a). Consistent with the above results, CXADR knockout did not affect the formation of spheres (Supplementary Fig. 14b), but the migration activity (Supplementary Fig. 14c) and the differentiation efficiency of postmigration NCSCs were considerably increased in the CXADR KO group compared to control DS cells (DS1 KO: 32.7 ± 1.5%, DS2 KO: 39.1 ± 2.5%, DS3 KO: 37.4 ± 3.8%; DS1: 17.9 ± 0.3%, DS2: 12.6 ± 3.7%, DS3: 12.7 ± 3.5%) (Supplementary Fig. 14d). The above data further demonstrate that CXADR triplication is involved in the migration defects of the DS-NCSCs.

## Discussion

In this study, DS-specific hiPSC lines were successfully reprogrammed from somatic cells with trisomy of HSA21 through overexpression of pluripotency factors. When induced to differentiate into neural crest progeny, DS cells presented a migration deficit phenotype and consequently severely impaired generation of the postmigratory neural crest. Further analysis revealed that the migration defects of the DS cells could be partially attributed to triplication of the CXADR gene on HSA21.

Developmental or functional anomalies of the neural crest are common in virtually all DS individuals, as was evident by the presence of characteristic craniofacial dysmorphology. Moreover, individuals with T21 have a 50- to 100-fold risk for HSCR compared with healthy individuals due to a lack of neural crest-derived enteric ganglion cells [27]. Neural crest defects could also result in various pathologies, including tooth abnormalities, PNS and SNS dysfunction, and cardiac cushion defects in some people with DS [28]. However, the underlying mechanism remains poorly understood. In recent years, disease-specific hiPSCs that show self-renewal and pluripotency have provided an invaluable tool for the study of disease modeling without ethical conflicts. Indeed, a large body of evidence indicates that DS-specific hiPSCs could be reprogrammed from adult fibroblasts or amniotic fluid cells using integrating (retrovirus or lentivirus) and nonintegrating methods (episomal vectors or Sendai virus). Most of these studies focused on the phenotypes of neurogenesis and hematopoiesis in DS [5]. Here, we successfully established 3 DS-hiPSC lines with the characteristic karyotype of 3 copies of HSA21 using Sendai viral vectors. Our study showed that the migratory ability and the generation of migrating NCSCs (p75^high^HNK1^+^ cells) from the DS-hiPSCs were significantly inhibited compared to those of the control cells in both monolayer and EB-based protocols, which was consistent with the results of a previous study using Ts65Dn mice [2]. Furthermore, our results showed that epithelial markers were significantly overexpressed, while the expression of mesenchymal-related genes was dramatically reduced in the neural crest derived from the DS group cells compared to the control group cells. These results suggest that the EMT process may be inhibited due to the triplication of genes in HSA21, thus leading to a lower number of migrating NCSCs derived from the DS-hiPSCs than the control hiPSCs. A previous study using Ts65Dn mice also revealed deficient mitosis of the neural crest in the pharyngeal arch [2]. However, we found that the proliferative potential of hiPSC-derived neural crest was similar between triploid and euploid cells. These conflicting findings may be partially due to the differences in genetic background, anatomy, physiology, and pathophysiology between humans and rodents.

Numerous dosage-sensitive genes on chromosome 21 have been identified to help elucidate the molecular mechanism underlying distinct DS phenotypes. The triplication of Ets2 was the first gene change that was identified as a cause of craniofacial deficits, since Ets2 transgenic mice presented DS-like skeletal abnormalities [29]. Another study, however, showed that deletion of the extra copy of Ets2 did not rescue the craniofacial phenotype in Ts65Dn mice [30]. Consequently, Ets2 may not be required for craniofacial abnormalities in DS [30]. Three copies of DYRK1A genes were also reported to be involved in DS-associated craniofacial abnormalities. Normalization of Dyrk1a copy number or treatment with a Dyrk1a inhibitor in trisomic Ts65Dn mice helped to normalize many dimensions of the cranial vault but did not correct all craniofacial abnormalities [31]. Furthermore, DYRK1A, DSCAM, and PAK1 were thought to be associated with cell migration defects in DS cells [26, 32]. In our study, we performed RNA sequencing to further determine the dosage-sensitive genes responsible for neurocristopathy in DS. The results indicated considerable differences in both genome-wide and chromosome 21 gene expression patterns between the DS-NCSCs and the control NCSCs. Unfortunately, we found that most of the above listed genes (DSCAM, SLC19A1, COL6A1, ETS2, PAK1, DYRK1A, and others) were barely expressed in NCSCs, or only mild changes in gene expression levels between the DS and control cells were detected.

Therefore, three dose-sensitive genes (CXADR, SUMO3, and COL18A1) located on chromosome 21 and significantly overexpressed in the DS-NCSCs were identified. These 3 genes have been reported to be involved in the migration of diverse cell types. To verify their role in neural crest migration, we modified the expression levels of these genes by shRNA-mediated gene knockdown or lentivirus overexpression in the DS-hiPSCs and the euploid hPSCs, respectively. Our results showed that only knockdown of CXADR, but not SUMO3 or COL18A1, could improve the migration of the DS-NCSCs and rescue the generation of migratory neural crest from the DS-hiPSCs. We also observed that cell migration was substantially impaired when CXADR expression was enhanced in the euploid NCSCs. Indeed, CXADR has been implicated in the pathogenesis of DS. Li et al. discovered an association between 21q21.1 microduplication of CXADR gene and developmental delay in DS individuals [33]. Palmer et al. also showed that CXADR was directly involved in neuronal cell–cell interactions and neurite outgrowth, and its expression was altered in both excitatory and inhibitory neuronal populations in the DS brain [34]. Accordingly, overexpression of CXADR might play a critical role in perturbation of the migration and development of the neural crest, thus contributing to craniofacial dysmorphology in DS.

In addition, disruption of SHH signaling has been implicated in neural crest migration defects, which in turn lead to craniofacial abnormalities and HSCR in Bardet-Biedl syndrome [35]. A previous study revealed that the expression of PAK1 was substantially increased in DS cells, and inhibition of the PAK1 pathway could significantly rescue the migration defects of DS GABAergic neurons [26]. In addition, it was reported that CXADR could regulate the stability and function of AKT inhibitors, and loss of CXADR could lead to hyperactivation of AKT and promote TGFβ1-induced EMT [23]. However, our results indicated that the protein levels of AKT and p-AKT were similar during neural crest development in hiPSCs between the DS group and the control group, while PAK1 inhibition or SHH stimulation could not rescue the migration defects in DS cells (data not shown). Therefore, the signaling pathway mediating neural crest deficits following CXADR overexpression in DS individuals needs further elucidation.

In conclusion, our research indicated that NCSCs derived from DS-hiPSCs could efficiently simulate the functional defects of their in vivo counterparts, which may be a powerful tool for uncovering the mechanisms underlying neural crest deficits, including ENS/PNS/SNS dysfunction, in DS individuals. The results also revealed that CXADR may play important roles in the pathogenesis of craniofacial deformities in DS.

## Supplementary information

Agreement from all authors.

Supplementary figure 1

Supplementary figure 2

Supplementary figure 3

Supplementary figure 4

Supplementary figure 5

Supplementary figure 6

Supplementary figure 7

Supplementary figure 8

Supplementary figure 9

Supplementary figure 10

Supplementary figure 11

Supplementary figure 12

Supplementary figure 13

Supplementary figure 14

Supplementary Figure legends

Supplementary Materials and Methods

Supplementary table 1

Supplementary table 2

Supplementary table 3

Supplementary table 4

Supplementary table 5

Supplementary table 6

Supplementary table 7

Supplementary table 8

Original western blots 1

Original western blots 2

Original western blots 3

## Data Availability

The authors declare that all data supporting the results in this study are available within the paper and its Supplementary Information. Raw data are available from the corresponding author upon reasonable request. The RNA-Seq data have been deposited in the GEO database under accession number GSE190305, and the NGDC database under accession number HRA003303. Source data are provided with this paper.
