# Supplementary material for: Migration deficits of the neural crest caused by CXADR triplication in a human Down syndrome stem cell model
Source: Cell Death Dis. 2022 Dec 5;13(12):1018. doi: 10.1038/s41419-022-05481-6 (PMC9722909; doi:10.1038/s41419-022-05481-6)
Supplement: Supplementary file 1 — Agreement from all authors. [file 41419_2022_5481_MOESM1_ESM.pdf]

主　题:	Re: mergeg PDF	
发件人:	"刘欢瑶" <601920081@qq.com>	2022-11-25 23:42:17
收件人:	"李伟强" <liweiq6@mail.sysu.edu.cn>	

yes，I agree.

---Original---

From: "李伟强"<liweiq6@mail.sysu.edu.cn>
Date: Fri, Nov 25, 2022 23:35 PM
To: "601920081"<601920081@qq.com>;"wwj0760"<wwj0760@163.com>;"huiyanwang1990"<huiyanwang1990@163.com>;"largestone_1984"<largestone_1984@163.com>;"zengjixiao"<zengjixiao@163.com>;"huangshanshan2333"<huangshanshan2333@163.com>;"chiuwch"<chiuwch@mail3.sysu.edu.cn>;"fanyubao1990"<fanyubao1990@163.com>;
Subject: mergeg PDF

Dear colleagues,

I am Weiqiang Li, the corresponding author of the manuscript CDDIS-22-0402RR, which is entitled with “Migration deficits of the neural crest caused by CXADR triplication in a human Down syndrome stem cell model”.

In this manuscript, Weijia Wang, Jiaqi Sun, and Yong Yuan were added as co-authors in the manuscript, since Weijia Wang (listed as a co-first author) and Yong Yuan (listed as a co-corresponding author) performed the experiments, analyzed the experimental data and drafted the manuscripts, while Jiaqi Sun (listed as a co-author) provided technical and material support during the manuscript was revised. Their contributions were clearly stated in the section of “Contributions” in the manuscript. The attached file was the latest version of the manuscript.

I wonder whether you agree with the changes in the author list. Thanks a lot.

Best,

Weiqiang Li

本邮件及其附件含有发送给特定个人和用于特定目的的信息。如果您不是预期的收件人，请立即删除本邮件并通知发件人。严禁任何非预期的收件人使用、传播、分发或复制本邮件或其附件。
This email and its attachments may contain confidential information intended for a specific individual and purpose. If you are not the intended recipient, you should delete this email and notify the sender immediately. Any use, dissemination, distribution, or copying of this email or its attachments by persons other than the intended recipient(s), is strictly prohibited.

主　题:	回复：mergeg PDF	
发件人:	"黄珊珊" <huangshanshan2333@163.com>	2022-11-25 23:39:43
收件人:	"李伟强老师" <liweiq6@mail.sysu.edu.cn>	

Yes，I agree.

---- 回复的原邮件 ----

发件人	李伟强<liweiq6@mail.sysu.edu.cn>
日期	2022年11月25日 23:35
收件人	601920081@qq.com<601920081@qq.com>、wwj0760@163.com<wwj0760@163.com>、huiyanwang1990@163.com<huiyanwang1990@163.com>、largestone_1984@163.com<largestone_1984@163.com>、zengjixiao@163.com<zengjixiao@163.com>、huangshanshan2333@163.com<huangshanshan2333@163.com>、chiuwch@mail3.sysu.edu.cn<chiuwch@mail3.sysu.edu.cn>、fanyubao1990@163.com<fanyubao1990@163.com>
主题	mergeg PDF

Dear colleagues,

I am Weiqiang Li, the corresponding author of the manuscript CDDIS-22-0402RR, which is entitled with “Migration deficits of the neural crest caused by CXADR triplication in a human Down syndrome stem cell model”.

In this manuscript, Weijia Wang, Jiaqi Sun, and Yong Yuan were added as co-authors in the manuscript, since Weijia Wang (listed as a co-first author) and Yong Yuan (listed as a co-corresponding author) performed the experiments, analyzed the experimental data and drafted the manuscripts, while Jiaqi Sun (listed as a co-author) provided technical and material support during the manuscript was revised. Their contributions were clearly stated in the section of “Contributions” in the manuscript. The attached file was the latest version of the manuscript.

I wonder whether you agree with the changes in the author list. Thanks a lot.

Best,

Weiqiang Li

本邮件及其附件含有发送给特定个人和用于特定目的的信息。如果您不是预期的收件人，请立即删除本邮件并通知发件人。严禁任何非预期的收件人使用、传播、分发或复制本邮件或其附件。
This email and its attachments may contain confidential information intended for a specific individual and purpose. If you are not the intended recipient, you should delete this email and notify the sender immediately. Any use, dissemination, distribution, or copying of this email or its attachments by persons other than the intended recipient(s), is strictly prohibited.

主　题:	Re:mergeg PDF	
发件人:	"王伟佳" <wwj0760@163.com>	2022-11-26 0:17:43
收件人:	"李伟强" <liweiq6@mail.sysu.edu.cn>	

Yes , I agree.　Weijia Wang

在 2022-11-25 23:35:22，"李伟强" <liweiq6@mail.sysu.edu.cn> 写道:

> Dear colleagues,
>
> I am Weiqiang Li, the corresponding author of the manuscript CDDIS-22-0402RR, which is entitled with “Migration deficits of the neural crest caused by CXADR triplication in a human Down syndrome stem cell model”.
>
> In this manuscript, Weijia Wang, Jiaqi Sun, and Yong Yuan were added as co-authors in the manuscript, since Weijia Wang (listed as a co-first author) and Yong Yuan (listed as a co-corresponding author) performed the experiments, analyzed the experimental data and drafted the manuscripts, while Jiaqi Sun (listed as a co-author) provided technical and material support during the manuscript was revised. Their contributions were clearly stated in the section of “Contributions” in the manuscript. The attached file was the latest version of the manuscript.
>
> I wonder whether you agree with the changes in the author list. Thanks a lot.
>
> Best,
>
> Weiqiang Li
>
> ---
>
> 本邮件及其附件含有发送给特定个人和用于特定目的的信息。如果您不是预期的收件人，请立即删除本邮件并通知发件人。严禁任何非预期的收件人使用、传播、分发或复制本邮件或其附件。
> This email and its attachments may contain confidential information intended for a specific individual and purpose. If you are not the intended recipient, you should delete this email and notify the sender immediately. Any use, dissemination, distribution, or copying of this email or its attachments by persons other than the intended recipient(s), is strictly prohibited.

主　题:	Re:mergeg PDF	
发件人:	huiyanwang1990 <huiyanwang1990@163.com>	2022-11-26 14:11:00
收件人:	"李伟强" <liweiq6@mail.sysu.edu.cn>	

Yes, I agree.

在 2022-11-25 23:35:22，"李伟强" <liweiq6@mail.sysu.edu.cn> 写道:

> Dear colleagues,
>
> I am Weiqiang Li, the corresponding author of the manuscript CDDIS-22-0402RR, which is entitled with “Migration deficits of the neural crest caused by CXADR triplication in a human Down syndrome stem cell model”.
>
> In this manuscript, Weijia Wang, Jiaqi Sun, and Yong Yuan were added as co-authors in the manuscript, since Weijia Wang (listed as a co-first author) and Yong Yuan (listed as a co-corresponding author) performed the experiments, analyzed the experimental data and drafted the manuscripts, while Jiaqi Sun (listed as a co-author) provided technical and material support during the manuscript was revised. Their contributions were clearly stated in the section of “Contributions” in the manuscript. The attached file was the latest version of the manuscript.
>
> I wonder whether you agree with the changes in the author list. Thanks a lot.
>
> Best,
>
> Weiqiang Li
>
> 本邮件及其附件含有发送给特定个人和用于特定目的的信息。如果您不是预期的收件人，请立即删除本邮件并通知发件人。严禁任何非预期的收件人使用、传播、分发或复制本邮件或其附件。
> This email and its attachments may contain confidential information intended for a specific individual and purpose. If you are not the intended recipient, you should delete this email and notify the sender immediately. Any use, dissemination, distribution, or copying of this email or its attachments by persons other than the intended recipient(s), is strictly prohibited.

主　题:	Re: mergeg PDF
发件人:	"hweijun@mail.sysu.edu.cn" <hweijun@mail.sysu.edu.cn> 2022-11-25 23:41:58
收件人:	"李伟强" <liweiq6@mail.sysu.edu.cn>

I agree with the changes in the author list.

Weijun Huang

On Fri, Nov 25, 2022 23:02 PM 李伟强<liweiq6@mail.sysu.edu.cn> wrote:

Dear colleagues,

I am Weiqiang Li, the corresponding author of the manuscript CDDIS-22-0402RR, which is entitled with "Migration deficits of the neural crest caused by CXADR triplication in a human Down syndrome stem cell model".

In this manuscript, Weijia Wang, Jiaqi Sun, and Yong Yuan were added as co-authors in the manuscript, since Weijia Wang (listed as a co-first author) and Yong Yuan (listed as a co-corresponding author) performed the experiments, analyzed the experimental data and drafted the manuscripts, while Jiaqi Sun (listed as a co-author) provided technical and material support during the manuscript was revised. Their contributions were clearly stated in the section of "Contributions" in the manuscript. The attached file was the latest version of the manuscript.

I wonder whether you agree with the changes in the author list. Thanks a lot.

Best,

Weiqiang Li

本邮件及其附件含有发送给特定个人和用于特定目的的信息。如果您不是预期的收件人，请立即删除本邮件并通知发件人。严禁任何非预期的收件人使用、传播、分发或复制本邮件或其附件。
This email and its attachments may contain confidential information intended for a specific individual and purpose. If you are not the intended recipient, you should delete this email and notify the sender immediately. Any use, dissemination, distribution, or copying of this email or its attachments by persons other than the intended recipient(s), is strictly prohibited.

主　题:	Re: mergeg PDF
发件人:	"翟志臣" <zhaizc@scut.edu.cn> 2022-11-26 13:08:53
收件人:	"李伟强" <liweiq6@mail.sysu.edu.cn>

Yes, I agree.

Best,

Zhichen Zhai

-----原始邮件-----
发件人:"李伟强" <liweiq6@mail.sysu.edu.cn>
发送时间:2022-11-25 23:02:04 (星期五)
收件人: 601920081@qq.com, huangshanshan2333@163.com, wwj0760@163.com, huiyanwang1990@163.com, hweijun@mail.sysu.edu.cn, zhaizc@scut.edu.cn, largestone_1984@163.com, fanyubao1990@163.com, sun_jiaqi@grmh-gdl.cn, lidairui@mail.sysu.edu.cn, chiuwch@mail3.sysu.edu.cn, laixq8@mail.sysu.edu.cn, zengjixiao@163.com, keqiong3@mail.sysu.edu.cn, wangt69@mail.sysu.edu.cn, xiangp@mail.sysu.edu.cn, yuany@zsph.com, zhxinch@mail.sysu.edu.cn
抄送:
主题: mergeg PDF

Dear colleagues,

I am Weiqiang Li, the corresponding author of the manuscript CDDIS-22-0402RR, which is entitled with “Migration deficits of the neural crest caused by CXADR triplication in a human Down syndrome stem cell model”.

In this manuscript, Weijia Wang, Jiaqi Sun, and Yong Yuan were added as co-authors in the manuscript, since Weijia Wang (listed as a co-first author) and Yong Yuan (listed as a co-corresponding author) performed the experiments, analyzed the experimental data and drafted the manuscripts, while Jiaqi Sun (listed as a co-author) provided technical and material support during the manuscript was revised. Their contributions were clearly stated in the section of “Contributions” in the manuscript. The attached file was the latest version of the manuscript.

I wonder whether you agree with the changes in the author list. Thanks a lot.

Best,

Weiqiang Li

本邮件及其附件含有发送给特定个人和用于特定目的的信息。如果您不是预期的收件人，请立即删除本邮件并通知发件人。严禁任何非预期的收件人使用、传播、分发或复制本邮件或其附件。
This email and its attachments may contain confidential information intended for a specific individual and purpose. If you are not the intended recipient, you should delete this email and notify the sender immediately. Any use, dissemination, distribution, or copying of this email or its attachments by persons other than the intended recipient(s), is strictly prohibited.

主　题:	Re: mergeg PDF	
发件人:	"Ding wang" <largestone_1984@163.com>	2022-11-26 15:33:17
收件人:	"李伟强" <liweiq6@mail.sysu.edu.cn>	

Yes，I agree.

---- Replied Message ----

From	李伟强<liweiq6@mail.sysu.edu.cn>
Date	11/25/2022 23:35
To	601920081<601920081@qq.com>,<br>wwj0760<wwj0760@163.com>,<br>huiyanwang1990<huiyanwang1990@163.com>,<br>largestone_1984<largestone_1984@163.com>,<br>zengjixiao<zengjixiao@163.com>,<br>huangshanshan2333<huangshanshan2333@163.com>,<br>chiuwch<chiuwch@mail3.sysu.edu.cn>,<br>fanyubao1990<fanyubao1990@163.com>
Subject	mergeg PDF

Dear colleagues,

I am Weiqiang Li, the corresponding author of the manuscript CDDIS-22-0402RR, which is entitled with “Migration deficits of the neural crest caused by CXADR triplication in a human Down syndrome stem cell model”.

In this manuscript, Weijia Wang, Jiaqi Sun, and Yong Yuan were added as co-authors in the manuscript, since Weijia Wang (listed as a co-first author) and Yong Yuan (listed as a co-corresponding author) performed the experiments, analyzed the experimental data and drafted the manuscripts, while Jiaqi Sun (listed as a co-author) provided technical and material support during the manuscript was revised. Their contributions were clearly stated in the section of “Contributions” in the manuscript. The attached file was the latest version of the manuscript.

I wonder whether you agree with the changes in the author list. Thanks a lot.

Best,

Weiqiang Li

本邮件及其附件含有发送给特定个人和用于特定目的的信息。如果您不是预期的收件人，请立即删除本邮件并通知发件人。严禁任何非预期的收件人使用、传播、分发或复制本邮件或其附件。
This email and its attachments may contain confidential information intended for a specific individual and purpose. If you are not the intended recipient, you should delete this email and notify the sender immediately. Any use, dissemination, distribution, or copying of this email or its attachments by persons other than the intended recipient(s), is strictly prohibited.

主　题:	Re: mergeg PDF	
发件人:	fanyubao1990 <fanyubao1990@163.com>	2022-11-26 8:36:38
收件人:	"liweiq6@mail.sysu.edu.cn" <liweiq6@mail.sysu.edu.cn>	

yes，I agree.

---- Replied Message ----

From	李伟强<liweiq6@mail.sysu.edu.cn>
Date	11/25/2022 23:35
To	601920081<601920081@qq.com>,<br>wwj0760<wwj0760@163.com>,<br>huiyanwang1990<huiyanwang1990@163.com>,<br>largestone_1984<largestone_1984@163.com>,<br>zengjixiao<zengjixiao@163.com>,<br>huangshanshan2333<huangshanshan2333@163.com>,<br>chiuwch<chiuwch@mail3.sysu.edu.cn>,<br>fanyubao1990<fanyubao1990@163.com>
Subject	mergeg PDF

Dear colleagues,

I am Weiqiang Li, the corresponding author of the manuscript CDDIS-22-0402RR, which is entitled with “Migration deficits of the neural crest caused by CXADR triplication in a human Down syndrome stem cell model”.

In this manuscript, Weijia Wang, Jiaqi Sun, and Yong Yuan were added as co-authors in the manuscript, since Weijia Wang (listed as a co-first author) and Yong Yuan (listed as a co-corresponding author) performed the experiments, analyzed the experimental data and drafted the manuscripts, while Jiaqi Sun (listed as a co-author) provided technical and material support during the manuscript was revised. Their contributions were clearly stated in the section of “Contributions” in the manuscript. The attached file was the latest version of the manuscript.

I wonder whether you agree with the changes in the author list. Thanks a lot.

Best,

Weiqiang Li

本邮件及其附件含有发送给特定个人和用于特定目的的信息。如果您不是预期的收件人，请立即删除本邮件并通知发件人。严禁任何非预期的收件人使用、传播、分发或复制本邮件或其附件。
This email and its attachments may contain confidential information intended for a specific individual and purpose. If you are not the intended recipient, you should delete this email and notify the sender immediately. Any use, dissemination, distribution, or copying of this email or its attachments by persons other than the intended recipient(s), is strictly prohibited.

主　题:	回复：mergeg PDF
发件人:	"孙佳琦" <sun_jiaqi@grmh-gdl.cn> 2022-11-26 9:52:12
收件人:	"李伟强" <liweiq6@mail.sysu.edu.cn>

Yes, I agree.

发件人：李伟强 <liweiq6@mail.sysu.edu.cn>
发送时间：2022年11月25日(星期五) 23:02
收件人：601920081 <601920081@qq.com>; huangshanshan2333 <huangshanshan2333@163.com>; wwj0760 <wwj0760@163.com>; huiyanwang1990 <huiyanwang1990@163.com>; hweijun <hweijun@mail.sysu.edu.cn>; zhaizc <zhaizc@scut.edu.cn>; largestone_1984 <largestone_1984@163.com>; fanyubao1990 <fanyubao1990@163.com>; 孙佳琦 <sun_jiaqi@grmh-gdl.cn>; lidairui <lidairui@mail.sysu.edu.cn>; chiuwch <chiuwch@mail3.sysu.edu.cn>; laixq8 <laixq8@mail.sysu.edu.cn>; zengjixiao <zengjixiao@163.com>; keqiong3 <keqiong3@mail.sysu.edu.cn>; wangt69 <wangt69@mail.sysu.edu.cn>; xiangp <xiangp@mail.sysu.edu.cn>; yuany <yuany@zsph.com>; zhxinch <zhxinch@mail.sysu.edu.cn>
主　题：mergeg PDF

Dear colleagues,

I am Weiqiang Li, the corresponding author of the manuscript CDDIS-22-0402RR, which is entitled with “Migration deficits of the neural crest caused by CXADR triplication in a human Down syndrome stem cell model”.

In this manuscript, Weijia Wang, Jiaqi Sun, and Yong Yuan were added as co-authors in the manuscript, since Weijia Wang (listed as a co-first author) and Yong Yuan (listed as a co-corresponding author) performed the experiments, analyzed the experimental data and drafted the manuscripts, while Jiaqi Sun (listed as a co-author) provided technical and material support during the manuscript was revised. Their contributions were clearly stated in the section of “Contributions” in the manuscript. The attached file was the latest version of the manuscript.

I wonder whether you agree with the changes in the author list. Thanks a lot.

Best,

Weiqiang Li

本邮件及其附件含有发送给特定个人和用于特定目的的信息。如果您不是预期的收件人，请立即删除本邮件并通知发件人。严禁任何非预期的收件人使用、传播、分发或复制本邮件或其附件。
This email and its attachments may contain confidential information intended for a specific individual and purpose. If you are not the intended recipient, you should delete this email and notify the sender immediately. Any use, dissemination, distribution, or copying of this email or its attachments by persons other than the intended recipient(s), is strictly prohibited.

主　题:	Re: mergeg PDF
发件人:	"lidairui@mail.sysu.edu.cn" <lidairui@mail.sysu.edu.cn> 2022-11-26 12:33:18
收件人:	"李伟强" <liweiq6@mail.sysu.edu.cn>

Yes, I agree

------------------ Original ------------------

From: 李伟强 <liweiq6@mail.sysu.edu.cn>
Date: Fri,Nov 25,2022 11:02 PM
To: 601920081 <601920081@qq.com>, huangshanshan2333 <huangshanshan2333@163.com>, wwj0760 <wwj0760@163.com>, huiyanwang1990 <huiyanwang1990@163.com>, hweijun <hweijun@mail.sysu.edu.cn>, zhaizc <zhaizc@scut.edu.cn>, largestone_1984 <largestone_1984@163.com>, fanyubao1990 <fanyubao1990@163.com>, sun_jiaqi <sun_jiaqi@grmh-gdl.cn>, lidairui <lidairui@mail.sysu.edu.cn>, chiuwch <chiuwch@mail3.sysu.edu.cn>, laixq8 <laixq8@mail.sysu.edu.cn>, zengjixiao <zengjixiao@163.com>, keqiong3 <keqiong3@mail.sysu.edu.cn>, wangt69 <wangt69@mail.sysu.edu.cn>, xiangp <xiangp@mail.sysu.edu.cn>, yuany <yuany@zsph.com>, zhxinch <zhxinch@mail.sysu.edu.cn>
Subject: Re: mergeg PDF

Dear colleagues,

I am Weiqiang Li, the corresponding author of the manuscript CDDIS-22-0402RR, which is entitled with "Migration deficits of the neural crest caused by CXADR triplication in a human Down syndrome stem cell model".

In this manuscript, Weijia Wang, Jiaqi Sun, and Yong Yuan were added as co-authors in the manuscript, since Weijia Wang (listed as a co-first author) and Yong Yuan (listed as a co-corresponding author) performed the experiments, analyzed the experimental data and drafted the manuscripts, while Jiaqi Sun (listed as a co-author) provided technical and material support during the manuscript was revised. Their contributions were clearly stated in the section of "Contributions" in the manuscript. The attached file was the latest version of the manuscript.

I wonder whether you agree with the changes in the author list. Thanks a lot.

Best,

Weiqiang Li

本邮件及其附件含有发送给特定个人和用于特定目的的信息。如果您不是预期的收件人，请立即删除本邮件并通知发件人。严禁任何非预期的收件人使用、传播、分发或复制本邮件或其附件。
This email and its attachments may contain confidential information intended for a specific individual and purpose. If you are not the intended recipient, you should delete this email and notify the sender immediately. Any use, dissemination, distribution, or copying of this email or its attachments by persons other than the intended recipient(s), is strictly prohibited.

主　题:	回复:mergeg PDF
发件人:	"邱暐程" <chiuwch@mail3.sysu.edu.cn> 2022-11-25 23:50:42
收件人:	"李伟强" <liweiq6@mail.sysu.edu.cn>

Yes, I agree
--------------原始邮件--------------
发件人："李伟强 "<liweiq6@mail.sysu.edu.cn>;
发送时间：2022年11月25日(星期五) 晚上11:35
收件人："601920081" <601920081@qq.com>;"wwj0760" <wwj0760@163.com>;"huiyanwang1990" <huiyanwang1990@163.com>;"largestone_1984" <largestone_1984@163.com>;"zengjixiao" <zengjixiao@163.com>;"huangshanshan2333" <huangshanshan2333@163.com>;"chiuwch" <chiuwch@mail3.sysu.edu.cn>;"fanyubao1990" <fanyubao1990@163.com>;
主题：mergeg PDF

Dear colleagues,

I am Weiqiang Li, the corresponding author of the manuscript CDDIS-22-0402RR, which is entitled with “Migration deficits of the neural crest caused by CXADR triplication in a human Down syndrome stem cell model”.

In this manuscript, Weijia Wang, Jiaqi Sun, and Yong Yuan were added as co-authors in the manuscript, since Weijia Wang (listed as a co-first author) and Yong Yuan (listed as a co-corresponding author) performed the experiments, analyzed the experimental data and drafted the manuscripts, while Jiaqi Sun (listed as a co-author) provided technical and material support during the manuscript was revised. Their contributions were clearly stated in the section of “Contributions” in the manuscript. The attached file was the latest version of the manuscript.

I wonder whether you agree with the changes in the author list. Thanks a lot.

Best,

Weiqiang Li

本邮件及其附件含有发送给特定个人和用于特定目的的信息。如果您不是预期的收件人，请立即删除本邮件并通知发件人。严禁任何非预期的收件人使用、传播、分发或复制本邮件或其附件。
This email and its attachments may contain confidential information intended for a specific individual and purpose. If you are not the intended recipient, you should delete this email and notify the sender immediately. Any use, dissemination, distribution, or copying of this email or its attachments by persons other than the intended recipient(s), is strictly prohibited.

主　题:	Re: mergeg PDF	
发件人:	"赖兴强" <laixq8@mail.sysu.edu.cn>	2022-11-26 13:03:51
收件人:	"李伟强" <liweiq6@mail.sysu.edu.cn>	

yes，I agree.

-----原始邮件-----
发件人:"李伟强" <liweiq6@mail.sysu.edu.cn>
发送时间:2022-11-25 23:02:04 (星期五)
收件人: 601920081@qq.com, huangshanshan2333@163.com, wwj0760@163.com, huiyanwang1990@163.com, hweijun@mail.sysu.edu.cn, zhaizc@scut.edu.cn, largestone_1984@163.com, fanyubao1990@163.com, sun_jiaqi@grmh-gdl.cn, lidairui@mail.sysu.edu.cn, chiuwch@mail3.sysu.edu.cn, laixq8@mail.sysu.edu.cn, zengjixiao@163.com, keqiong3@mail.sysu.edu.cn, wangt69@mail.sysu.edu.cn, xiangp@mail.sysu.edu.cn, yuany@zsph.com, zhxinch@mail.sysu.edu.cn
抄送:
主题: mergeg PDF

Dear colleagues,

I am Weiqiang Li, the corresponding author of the manuscript CDDIS-22-0402RR, which is entitled with "Migration deficits of the neural crest caused by CXADR triplication in a human Down syndrome stem cell model".

In this manuscript, Weijia Wang, Jiaqi Sun, and Yong Yuan were added as co-authors in the manuscript, since Weijia Wang (listed as a co-first author) and Yong Yuan (listed as a co-corresponding author) performed the experiments, analyzed the experimental data and drafted the manuscripts, while Jiaqi Sun (listed as a co-author) provided technical and material support during the manuscript was revised. Their contributions were clearly stated in the section of "Contributions" in the manuscript. The attached file was the latest version of the manuscript.

I wonder whether you agree with the changes in the author list. Thanks a lot.

Best,

Weiqiang Li

本邮件及其附件含有发送给特定个人和用于特定目的的信息。如果您不是预期的收件人，请立即删除本邮件并通知发件人。严禁任何非预期的收件人使用、传播、分发或复制本邮件或其附件。
This email and its attachments may contain confidential information intended for a specific individual and purpose. If you are not the intended recipient, you should delete this email and notify the sender immediately. Any use, dissemination, distribution, or copying of this email or its attachments by persons other than the intended recipient(s), is strictly prohibited.

本邮件及其附件含有发送给特定个人和用于特定目的的信息。如果您不是预期的收件人，请立即删除本邮件并通知发件人。严禁任何非预期的收件人使用、传播、分发或复制本邮件或其附件。
This email and its attachments may contain confidential information intended for a specific individual and purpose. If you are not the intended recipient, you should delete this email and notify the sender immediately. Any use, dissemination, distribution, or copying of this email or its attachments by persons other than the intended recipient(s), is strictly prohibited.

主　题:	Re: mergeg PDF	
发件人:	"曾纪晓" <zengjixiao@163.com>	2022-11-25 23:59:56
收件人:	"李伟强" <liweiq6@mail.sysu.edu.cn>	
附　件:	mergeg PDF.pdf	

Yes，I agree。

---- Replied Message ----

From	李伟强<liweiq6@mail.sysu.edu.cn>
Date	11/25/2022 23:35
To	601920081<601920081@qq.com>, wwj0760<wwj0760@163.com>, huiyanwang1990<huiyanwang1990@163.com>, largestone_1984<largestone_1984@163.com>, zengjixiao<zengjixiao@163.com>, huangshanshan2333<huangshanshan2333@163.com>, chiuwch<chiuwch@mail3.sysu.edu.cn>, fanyubao1990<fanyubao1990@163.com>
Subject	mergeg PDF

Dear colleagues,

I am Weiqiang Li, the corresponding author of the manuscript CDDIS-22-0402RR, which is entitled with “Migration deficits of the neural crest caused by CXADR triplication in a human Down syndrome stem cell model”.

In this manuscript, Weijia Wang, Jiaqi Sun, and Yong Yuan were added as co-authors in the manuscript, since Weijia Wang (listed as a co-first author) and Yong Yuan (listed as a co-corresponding author) performed the experiments, analyzed the experimental data and drafted the manuscripts, while Jiaqi Sun (listed as a co-author) provided technical and material support during the manuscript was revised. Their contributions were clearly stated in the section of “Contributions” in the manuscript. The attached file was the latest version of the manuscript.

I wonder whether you agree with the changes in the author list. Thanks a lot.

Best,

Weiqiang Li

本邮件及其附件含有发送给特定个人和用于特定目的的信息。如果您不是预期的收件人，请立即删除本邮件并通知发件人。严禁任何非预期的收件人使用、传播、分发或复制本邮件或其附件。
This email and its attachments may contain confidential information intended for a specific individual and purpose. If you are not the intended recipient, you should delete this email and notify the sender immediately. Any use, dissemination, distribution, or copying of this email or its attachments by persons other than the intended recipient(s), is strictly prohibited.

主　题:	Re: mergeg PDF
发件人:	keqiong3 <keqiong3@mail.sysu.edu.cn> 2022-11-26 4:45:18
收件人:	"李伟强" <liweiq6@mail.sysu.edu.cn>

Yes，I agree.

Qiong Ke

Center for stem cell biology and tissue engineering,
Sun Yat-sen University,
74#, Zhongshan Road 2 Guangzhou,
Guangdong 510080 P.R.China
Tel:86-20-87335982; Fax: 86-20-87335858

---- Replied Message ----

From	李伟强<liweiq6@mail.sysu.edu.cn>
Date	11/25/2022 23:02
To	<601920081@qq.com> , <huangshanshan2333@163.com> , <wwj0760@163.com> , <huiyanwang1990@163.com> , <hweijun@mail.sysu.edu.cn> , <zhaizc@scut.edu.cn> , <largestone_1984@163.com> , <fanyubao1990@163.com> , <sun_jiaqi@grmh-gdl.cn> , <lidairui@mail.sysu.edu.cn> , <chiuwch@mail3.sysu.edu.cn> , <laixq8@mail.sysu.edu.cn> , <zengjixiao@163.com> , <keqiong3@mail.sysu.edu.cn> , <wangt69@mail.sysu.edu.cn> , <xiangp@mail.sysu.edu.cn> , <yuany@zsph.com> , <zhxinch@mail.sysu.edu.cn>
Subject	mergeg PDF

Dear colleagues,

I am Weiqiang Li, the corresponding author of the manuscript CDDIS-22-0402RR, which is entitled with “Migration deficits of the neural crest caused by CXADR triplication in a human Down syndrome stem cell model”.

In this manuscript, Weijia Wang, Jiaqi Sun, and Yong Yuan were added as co-authors in the manuscript, since Weijia Wang (listed as a co-first author) and Yong Yuan (listed as a co-corresponding author) performed the experiments, analyzed the experimental data and drafted the manuscripts, while Jiaqi Sun (listed as a co-author) provided technical and material support during the manuscript was revised. Their contributions were clearly stated in the section of “Contributions” in the manuscript. The attached file was the latest version of the manuscript.

I wonder whether you agree with the changes in the author list. Thanks a lot.

Best,

Weiqiang Li

本邮件及其附件含有发送给特定个人和用于特定目的的信息。如果您不是预期的收件人，请立即删除本邮件并通知发件人。严禁任何非预期的收件人使用、传播、分发或复制本邮件或其附件。
This email and its attachments may contain confidential information intended for a specific individual and purpose. If you are not the intended recipient, you should delete this email and notify the sender immediately. Any use, dissemination, distribution, or copying of this email or its attachments by persons other than the intended recipient(s), is strictly prohibited.

主　题:	Re: mergeg PDF
发件人:	"王涛" <wangt69@mail.sysu.edu.cn> 2022-11-26 12:59:32
收件人:	"李伟强" <liweiq6@mail.sysu.edu.cn>

Yes, I agree!

-----原始邮件-----
发件人:"李伟强" <liweiq6@mail.sysu.edu.cn>
发送时间:2022-11-25 23:02:04 (星期五)
收件人: 601920081@qq.com, huangshanshan2333@163.com, wwj0760@163.com, huiyanwang1990@163.com, hweijun@mail.sysu.edu.cn, zhaizc@scut.edu.cn, largestone_1984@163.com, fanyubao1990@163.com, sun_jiaqi@grmh-gdl.cn, lidairui@mail.sysu.edu.cn, chiuwch@mail3.sysu.edu.cn, laixq8@mail.sysu.edu.cn, zengjixiao@163.com, keqiong3@mail.sysu.edu.cn, wangt69@mail.sysu.edu.cn, xiangp@mail.sysu.edu.cn, yuany@zsph.com, zhxinch@mail.sysu.edu.cn
抄送:
主题: mergeg PDF

Dear colleagues,

I am Weiqiang Li, the corresponding author of the manuscript CDDIS-22-0402RR, which is entitled with "Migration deficits of the neural crest caused by CXADR triplication in a human Down syndrome stem cell model".

In this manuscript, Weijia Wang, Jiaqi Sun, and Yong Yuan were added as co-authors in the manuscript, since Weijia Wang (listed as a co-first author) and Yong Yuan (listed as a co-corresponding author) performed the experiments, analyzed the experimental data and drafted the manuscripts, while Jiaqi Sun (listed as a co-author) provided technical and material support during the manuscript was revised. Their contributions were clearly stated in the section of "Contributions" in the manuscript. The attached file was the latest version of the manuscript.

I wonder whether you agree with the changes in the author list. Thanks a lot.

Best,

Weiqiang Li

本邮件及其附件含有发送给特定个人和用于特定目的的信息。如果您不是预期的收件人，请立即删除本邮件并通知发件人。严禁任何非预期的收件人使用、传播、分发或复制本邮件或其附件。
This email and its attachments may contain confidential information intended for a specific individual and purpose. If you are not the intended recipient, you should delete this email and notify the sender immediately. Any use, dissemination, distribution, or copying of this email or its attachments by persons other than the intended recipient(s), is strictly prohibited.

本邮件及其附件含有发送给特定个人和用于特定目的的信息。如果您不是预期的收件人，请立即删除本邮件并通知发件人。严禁任何非预期的收件人使用、传播、分发或复制本邮件或其附件。
This email and its attachments may contain confidential information intended for a specific individual and purpose. If you are not the intended recipient, you should delete this email and notify the sender immediately. Any use, dissemination, distribution, or copying of this email or its attachments by persons other than the intended recipient(s), is strictly prohibited.

主　题:	Re: mergeg PDF
发件人:	"项鹏" <xiangp@mail.sysu.edu.cn> 2022-11-26 16:51:04
收件人:	"李伟强" <liweiq6@mail.sysu.edu.cn>

Copy that and agree.

Andy Peng Xiang

-----原始邮件-----
发件人:"李伟强" <liweiq6@mail.sysu.edu.cn>
发送时间:2022-11-25 23:02:04 (星期五)
收件人: 601920081@qq.com, huangshanshan2333@163.com, wwj0760@163.com, huiyanwang1990@163.com, hweijun@mail.sysu.edu.cn, zhaizc@scut.edu.cn, largestone_1984@163.com, fanyubao1990@163.com, sun_jiaqi@grmh-gdl.cn, lidairui@mail.sysu.edu.cn, chiuwch@mail3.sysu.edu.cn, laixq8@mail.sysu.edu.cn, zengjixiao@163.com, keqiong3@mail.sysu.edu.cn, wangt69@mail.sysu.edu.cn, xiangp@mail.sysu.edu.cn, yuany@zsph.com, zhxinch@mail.sysu.edu.cn
抄送:
主题: mergeg PDF

Dear colleagues,

I am Weiqiang Li, the corresponding author of the manuscript CDDIS-22-0402RR, which is entitled with "Migration deficits of the neural crest caused by CXADR triplication in a human Down syndrome stem cell model".

In this manuscript, Weijia Wang, Jiaqi Sun, and Yong Yuan were added as co-authors in the manuscript, since Weijia Wang (listed as a co-first author) and Yong Yuan (listed as a co-corresponding author) performed the experiments, analyzed the experimental data and drafted the manuscripts, while Jiaqi Sun (listed as a co-author) provided technical and material support during the manuscript was revised. Their contributions were clearly stated in the section of "Contributions" in the manuscript. The attached file was the latest version of the manuscript.

I wonder whether you agree with the changes in the author list. Thanks a lot.

Best,

Weiqiang Li

本邮件及其附件含有发送给特定个人和用于特定目的的信息。如果您不是预期的收件人，请立即删除本邮件并通知发件人。严禁任何非预期的收件人使用、传播、分发或复制本邮件或其附件。
This email and its attachments may contain confidential information intended for a specific individual and purpose. If you are not the intended recipient, you should delete this email and notify the sender immediately. Any use, dissemination, distribution, or copying of this email or its attachments by persons other than the intended recipient(s), is strictly prohibited.

本邮件及其附件含有发送给特定个人和用于特定目的的信息。如果您不是预期的收件人，请立即删除本邮件并通知发件人。严禁任何非预期的收件人使用、传播、分发或复制本邮件或其附件。
This email and its attachments may contain confidential information intended for a specific individual and purpose. If you are not the intended recipient, you should delete this email and notify the sender immediately. Any use, dissemination, distribution, or copying of this email or its attachments by persons other than the intended recipient(s), is strictly prohibited.

主　题:	Re:mergeg PDF	
发件人:	"袁勇" <yuany@zsph.com>	2022-11-26 0:19:59
收件人:	"李伟强" <liweiq6@mail.sysu.edu.cn>	

Yes , I agree.　Yong Yuan

袁勇 zsph.com
默认部门 yuany@zsph.com

---- 原邮件信息 -----
发件人:李伟强<liweiq6@mail.sysu.edu.cn>
收件人:601920081@qq.com; huangshanshan2333@163.com; wwj0760@163.com; huiyanwang1990@163.com; hweijun@mail.sysu.edu.cn; zhaizc@scut.edu.cn; largestone_1984@163.com; fanyubao1990@163.com; sun_jiaqi@grmh-gdl.cn; lidairui@mail.sysu.edu.cn; chiuwch@mail3.sysu.edu.cn; laixq8@mail.sysu.edu.cn; zengjixiao@163.com; keqiong3@mail.sysu.edu.cn; wangt69@mail.sysu.edu.cn; xiangp@mail.sysu.edu.cn; yuany@zsph.com; zhxinch@mail.sysu.edu.cn;
发送时间:2022-11-25 23:02:43
主题:mergeg PDF

Dear colleagues,

I am Weiqiang Li, the corresponding author of the manuscript CDDIS-22-0402RR, which is entitled with “Migration deficits of the neural crest caused by CXADR triplication in a human Down syndrome stem cell model”.

In this manuscript, Weijia Wang, Jiaqi Sun, and Yong Yuan were added as co-authors in the manuscript, since Weijia Wang (listed as a co-first author) and Yong Yuan (listed as a co-corresponding author) performed the experiments, analyzed the experimental data and drafted the manuscripts, while Jiaqi Sun (listed as a co-author) provided technical and material support during the manuscript was revised. Their contributions were clearly stated in the section of “Contributions” in the manuscript. The attached file was the latest version of the manuscript.

I wonder whether you agree with the changes in the author list. Thanks a lot.

Best,

Weiqiang Li

本邮件及其附件含有发送给特定个人和用于特定目的的信息。如果您不是预期的收件人，请立即删除本邮件并通知发件人。严禁任何非预期的收件人使用、传播、分发或复制本邮件或其附件。
This email and its attachments may contain confidential information intended for a specific individual and purpose. If you are not the intended recipient, you should delete this email and notify the sender immediately. Any use, dissemination, distribution, or copying of this email or its attachments by persons other than the intended recipient(s), is strictly prohibited.

主　题:	Re: mergeg PDF	
发件人:	"张新春" <zhxinch@mail.sysu.edu.cn>	2022-11-26 15:59:32
收件人:	"李伟强" <liweiq6@mail.sysu.edu.cn>	

Prof Li,

I received your mail about "change author list".

Yes, I agree with the changes in the author list.

Best wishes.

Xinchun Zhang

-----原始邮件----.
发件人:"李伟强" <liweiq6@mail.sysu.edu.cn>
发送时间:2022-11-25 23:02:04 (星期五)
收件人: 601920081@qq.com, huangshanshan2333@163.com, wwj0760@163.com, huiyanwang1990@163.com, hweijun@mail.sysu.edu.cn, zhaizc@scut.edu.cn, largestone_1984@163.com, fanyubao1990@163.com, sun_jiaqi@grmh-gdl.cn, lidairui@mail.sysu.edu.cn, chiuwch@mail3.sysu.edu.cn, laixq8@mail.sysu.edu.cn, zengjixiao@163.com, keqiong3@mail.sysu.edu.cn, wangt69@mail.sysu.edu.cn, xiangp@mail.sysu.edu.cn, yuany@zsph.com, zhxinch@mail.sysu.edu.cn
抄送:
主题: mergeg PDF

Dear colleagues,

I am Weiqiang Li, the corresponding author of the manuscript CDDIS-22-0402RR, which is entitled with “Migration deficits of the neural crest caused by CXADR triplication in a human Down syndrome stem cell model”.

In this manuscript, Weijia Wang, Jiaqi Sun, and Yong Yuan were added as co-authors in the manuscript, since Weijia Wang (listed as a co-first author) and Yong Yuan (listed as a co-corresponding author) performed the experiments, analyzed the experimental data and drafted the manuscripts, while Jiaqi Sun (listed as a co-author) provided technical and material support during the manuscript was revised. Their contributions were clearly stated in the section of “Contributions” in the manuscript. The attached file was the latest version of the manuscript.

I wonder whether you agree with the changes in the author list. Thanks a lot.

Best,

Weiqiang Li

本邮件及其附件含有发送给特定个人和用于特定目的的信息。如果您不是预期的收件人，请立即删除本邮件并通知发件人。严禁任何非预期的收件人使用、传播、分发或复制本邮件或其附件。
This email and its attachments may contain confidential information intended for a specific individual and purpose. If you are not the intended recipient, you should delete this email and notify the sender immediately. Any use, dissemination, distribution, or copying of this email or its attachments by persons other than the intended recipient(s), is strictly prohibited.

本邮件及其附件含有发送给特定个人和用于特定目的的信息。如果您不是预期的收件人，请立即删除本邮件并通知发件人。严禁任何非预期的收件人使用、传播、分发或复制本邮件或其附件。
This email and its attachments may contain confidential information intended for a specific individual and purpose. If you are not the intended recipient, you should delete this email and notify the sender immediately. Any use, dissemination, distribution, or copying of this email or its attachments by persons other than the intended recipient(s), is strictly prohibited.
