# Supplementary material for: Migration deficits of the neural crest caused by CXADR triplication in a human Down syndrome stem cell model
Source: Cell Death Dis. 2022 Dec 5;13(12):1018. doi: 10.1038/s41419-022-05481-6 (PMC9722909; doi:10.1038/s41419-022-05481-6)
Supplement: Supplementary file 16 — Supplementary Figure legends [file 41419_2022_5481_MOESM16_ESM.docx]

**Supplementary Figures**

**Supplementary Fig. 1 Characterization of DS-hiPSCs**

1. The morphology of DS-hiPSCs. Scale bar: 250 μm.
2. The karyotype analysis of DS-hiPSCs.
3. The expression of pluripotency markers (OCT4, NANOG, SSEA4, and TRA-1-81) in DS-hiPSCs was detected by immunostaining. Scale bar: 100 μm.
4. The expression of an ectodermal marker (TUBB3), a mesodermal progenitor marker (TBXT), and an endodermal progenitor marker (SOX17) in differentiated DS-hiPSCs was detected by immunostaining. Scale bar: 100 μm.
5. HE staining showed that teratomas formed by DS-hiPSCs contained ectoderm-derived neural tubes, mesoderm-derived cartilage, and endoderm-derived glandular epithelium. Scale bar: 250 μm.

**Supplementary Fig. 2 The cell proliferation and apoptosis rate of day 7 differentiated cells were detected.**

1. The expression of Ki67 was detected by immunostaining. Scale bar: 100 μm.
2. The percentage of Ki67-labeled cells was calculated and compared between the control group and the DS group.
3. The proliferation of trisomic and euploid cells was assessed by CCK-8 assays.
4. Cell apoptosis was detected by anti-active caspase 3 staining and compared between the control group and the DS group. Scale bar: 100 μm.
5. Cell apoptosis was also detected by TUNEL assay and compared between the control group and the DS group. Scale bar: 100 μm.

Data are presented as the mean ± SD of three independent experiments. n=3. *P<0.05, **P<0.01, ***P<0.001, ****P<0.0001, one-way ANOVA.

**Supplementary Fig. 3 Migration defects of the neural crest derived from DS3-hiPSCs.**

1. Day 7 differentiated cells derived from AFC and DS3 hiPSCs formed spheres when cultured in suspension for 24 hours. n=50.
2. The expression of a neural crest-specific marker (SOX10) in differentiated cells of the DS3 group and the AFC group was detected by immunostaining.
3. Cells were observed under a phase-contrast microscope at 24 hours after attachment of spheres and cell migration area was calculated. n=9.
4. qPCR assay for the expression of epithelial markers (ECAD, CLDN7), premigratory markers (MSX2, DLX5, SOX9), and postmigratory markers (p75, SOX10, SNAI2) in day 7 cells of DS3 and AFC group. n=3.

Data are presented as the mean ± SD of three independent experiments. *P<0.05, **P<0.01, ***P<0.001, ****P<0.0001, one-way ANOVA (a) and two-tailed unpaired Student’s t test (c, d). Scale bar: 250 μm.

**Supplementary Fig. 4 Migration defects of the DS-NCSCs were verified by an EB-based differentiation protocol.**

1. Strategy for neural crest differentiation from hPSCs by the EB formation protocol.
2. The morphology of EBs formed in Aggrewell plates. Scale bar: 250 μm.
3. Morphology of EBs cultured in suspension in petri dishes. Scale bar: 250 μm.
4. Quantification and comparison of the diameters of EBs derived from the DS group and the control group. n=22.
5. The differentiated cells were observed under a phase-contrast microscope when EBs were attached for 24 hours and 48 hours. Scale bar: 250 μm.
6. The cell migration area at 24 hours and 48 hours after attachment was calculated and compared between the DS group and the control group. n=10.
7. The increased migration area between 24 hours and 48 hours after attachment was calculated and compared between the DS group and the control group. n=10
8. The migration distance (μm) of EB-derived cells was recorded by time-lapse imaging. n=20.
9. The migration velocity (μm per 30 min) of EB-derived cells was analyzed by time-lapse imaging. n=20.

Data are presented as the mean ± SD of three independent experiments. *P<0.05, **P<0.01, ***P<0.001, ****P<0.0001, two-tailed unpaired Student’s t test.

**Supplementary Fig. 5 Generation of migrating NCSCs by the monolayer differentiation protocol was inhibited in the DS3 group.**

1. The percentage of postmigratory NCSCs (p75^high^/HNK1^+^) induced with monolayer differentiation protocol was detected by FACS.
2. Enriched NCSCs maintained typical neural crest morphology during *in vitro* adherent culture.
3. The expression of a neural crest-specific marker (SOX10, p75, HNK1) in NCSCs was detected by immunostaining.
4. The expression of cranial neural crest-specific markers (ETS1, HOXA1 and LHX5) in NCSCs was detected by qPCR.

Data are presented as the mean ± SD of three independent experiments. n=3. *P<0.05, **P<0.01, ***P<0.001, ****P<0.0001, two-tailed unpaired Student’s t test. Scale bar: 250 μm.

**Supplementary Fig. 6 Generation of migrating NCSCs by an EB-based differentiation protocol was also impaired in the DS group.**

1. Postmigratory NCSCs (HNK1^+^/p75^high^) generated through EB-based protocol were measured and isolated by FACS.
2. Enriched NCSCs were observed under a phase-contrast microscope during *in vitro* adherent culture. Scale bar: 250 μm.
3. The expression of a neural crest-specific marker (SOX10, p75, HNK1) in NCSCs was detected by immunostaining. Scale bar: 100 μm.

Data are presented as the mean ± SD of three independent experiments. n=3. *P<0.05, **P<0.01, ***P<0.001, ****P<0.0001, two-tailed unpaired Student’s t test.

**Supplementary Fig. 7 Neuronal and Schwann cell differentiation of NCSCs.**

1. The expression of neural-specific markers (PRPH and TUBB3) in differentiated cells was detected by immunostaining. Scale bar: 100 μm.
2. The expression of Schwann-specific markers (GFAP and S100B) in differentiated cells was detected by immunostaining. Scale bar: 100 μm.

**Supplementary Fig. 8 Mesenchymal lineage differentiation of NCSCs.**

1. Mesenchymal-like cells emerged after exposure to MesenCult^TM^-ACF Plus Medium for 3–4 weeks. Scale bar: 250 μm.
2. FACS analysis for the detection of typical MSC surface markers in NCSC-derived mesenchymal-like cells.
3. Alizarin Red S staining, Oil Red O staining, and toluidine blue staining were used to analyze the osteogenic, adipogenic, and chondrogenic differentiation capacity of NCSC-derived mesenchymal-like cells, respectively. Scale bar: 250 μm.

**Supplementary Fig. 9 IPA analysis for the expression of genes associated with “cell movement” and “adhesion”.**

1. The top 20 genes with upregulated and downregulated expression related to “cell movement” in DS-NCSCs were analyzed by IPA software.
2. The top 20 genes with upregulated and downregulated expression related to “adhesion” in DS-NCSCs were analyzed by IPA software.

**Supplementary Fig. 10 The expression of CXADR, COL18A1, and SUMO3 in DS3-NCSCs was upregulated compared to that in AFC-NCSCs.**

1. qPCR assay for CXADR, COL18A1, and SUMO3 expression in day 7 differentiated cells during neural crest commitment in AFC and DS3.
2. qPCR assay for CXADR, COL18A1, and SUMO3 expression in AFC- and DS3-NCSCs enriched by FACS.
3. Protein levels of CXADR, COL18A1 and SUMO3 were evaluated in AFC- and DS3-NCSCs using western blotting.

Data are presented as the mean ± SD of three independent experiments. n=3. *P<0.05, **P<0.01, ***P<0.001, ****P<0.0001, two-tailed unpaired Student’s t test.

**Supplementary Fig. 11 Knockdown of COL18A1 and SUMO3 did not substantially improve the generation of migrating NCSCs from DS-hiPSCs.**

1. qPCR assay for COL18A1 and SUMO3 expression in DS1-hiPSCs after shRNA lentivirus transduction.
2. qPCR assay for COL18A1 and SUMO3 expression in DS2-hiPSCs after shRNA lentivirus transduction.
3. Protein expression of COL18A1 and SUMO3 in shRNA lentivirus-transduced DS1-hiPSCs was evaluated using western blotting.
4. Protein expression of COL18A1 and SUMO3 in shRNA lentivirus-transduced DS2-hiPSCs was evaluated using western blotting.
5. Postmigratory NCSCs (HNK1^+^/p75^high^) derived from shRNA-transduced DS1-hiPSCs were measured by FACS.
6. Postmigratory NCSCs (HNK1^+^/p75^high^) derived from shRNA-transduced DS2-hiPSCs were measured by FACS.

Data are presented as the mean ± SD of three independent experiments. n=3. *P<0.05, **P<0.01, ***P<0.001, ****P<0.0001, two-tailed unpaired Student’s t test.

**Supplementary Fig. 12 CXADR expression affects migration of AFC- and DS3-NCSCs.**

1. qPCR and western blotting assays for CXADR expression in nontarget (shCtrl) and CXADR knockdown (shCXADR) DS3 cells.
2. Spheres were formed by day 7 differentiated cells derived from AFC, DS3 shCtrl and DS3 shCXADR groups. n=50.
3. Cells were observed under a phase-contrast microscope at 24 hours after attachment of spheres and cell migration area was calculated. n=12.
4. Postmigratory NCSCs (p75^high^/HNK1^+^) derived from AFC, DS3 shCtrl and DS3 shCXADR groups were measured by FACS. n=3.
5. qPCR and western blotting assays for CXADR expression in AFC-hPSCs before and after lentiviral overexpression (AFC Ctrl vs. AFC CXADR).
6. Spheres were formed by day 7 differentiated cells derived from DS3, AFC Ctrl and AFC CXADR groups. n=50.
7. Cells were observed under a phase-contrast microscope at 24 hours after attachment of spheres and cell migration area was calculated. n=12.
8. Postmigratory NCSCs (p75^high^/HNK1^+^) derived from DS3, AFC Ctrl and AFC CXADR groups were measured by FACS. n=3.

Data are presented as the mean ± SD of three independent experiments. *P<0.05, **P<0.01, ***P<0.001, ****P<0.0001, one-way ANOVA (c, f) and two-tailed unpaired Student’s t test (a, b, d, e, g, h). Scale bar: 250 μm.

**Supplementary Fig. 13 IPA analysis of the differentially expressed genes in NCSCs derived from DS shCXADR group compared to DS shCtrl group.**

1. IPA functional annotation of all differentially expressed mRNAs. The dot plot of partially enriched functions. The color intensity of the nodes indicates the degree of IPA function enrichment. The horizontal axis indicates the gene ratio as the proportion of differentially expressed genes in the whole gene set. The size represents the number counts in a certain function.
2. IPA canonical pathway analysis of all differentially expressed mRNAs. The dot plot of partially enriched pathways. The color intensity of the nodes indicates the degree of canonical IPA pathway enrichment. The horizontal axis indicates the gene ratio as the proportion of differentially expressed genes in the whole gene set. The size represents the number counts in a certain pathway.

**Supplementary Fig. 14 CXADR knockout using CRISPR/Cas9 technology improved migration ability of DS-NCSCs.**

1. qPCR and western blotting assays for CXADR expression in wild type DS (DS1 WT, DS2 WT, DS3 WT) and CXADR-knockout DS (DS1 KO, DS2 KO, DS3 KO) groups. n=3.
2. Spheres were formed by day 7 differentiated cells derived from DS WT and DS KO groups. n=50.
3. Spheres formed by DS WT and DS KO groups were attached for 24 hours and cell migrating area was calculated. n=12.
4. Postmigratory NCSCs (p75^high^/HNK1^+^) derived from DS WT and DS KO groups were measured by FACS. n=3.

Data are presented as the mean ± SD of three independent experiments. *P<0.05, **P<0.01, ***P<0.001, ****P<0.0001, one-way ANOVA (d) and two-tailed unpaired Student’s t test (a, b, c, e, f). Scale bar: 250 μm.
