# Supplementary material for: Migration deficits of the neural crest caused by CXADR triplication in a human Down syndrome stem cell model
Source: Cell Death Dis. 2022 Dec 5;13(12):1018. doi: 10.1038/s41419-022-05481-6 (PMC9722909; doi:10.1038/s41419-022-05481-6)
Supplement: Supplementary file 17 — Supplementary Materials and Methods [file 41419_2022_5481_MOESM17_ESM.docx]

**Supplementary Materials and Methods**

**Human ESC/iPSC culture**

DS-specific hiPSC lines derived from human amniotic fluid cells or embryonic fibroblasts with trisomy of HSA21 (DS1, DS2, DS3) and wild-type hPSC lines, including H9 (a hESC line), human embryonic fibroblast-derived hiPSCs (HEF-hiPSCs; designated HEF), and amniotic fluid cell-derived hiPSCs (AFC-hiPSCs) [1], were used in this study. All procedures performed in the studies involving human participants were conducted in accordance with the standards of the Ethics Committee of Sun Yat-Sen University and with the 1964 Helsinki Declaration and its later amendments or comparable ethical standards. Cells were cultured in mTeSR^TM^1 (StemCell Technologies, Vancouver, Canada) on Matrigel Matrix (BD Biosciences, San Diego, CA, USA)-coated plates. The culture medium was completely replenished every day, and cells were passaged every 4–5 days using Accutase (Invitrogen, Carlsbad, CA, USA).

**Cell reprogramming**

DS-specific hiPSCs were generated by overexpression of the pluripotency markers OCT4, SOX2, KLF4, and cMYC in human embryonic fibroblasts (DS1) or amniotic fluid cells (DS2, DS3) with T21 using Sendai viral vectors (Thermo Fisher Scientific, Rutherford, NJ, USA) according to the manufacturer’s instructions. All procedures performed in studies involving human tissues or cells were conducted in accordance with the standards of the Ethics Committee of Zhongshan School of Medicine, Sun Yat-sen University.

For determination of the *in vitro* differentiation capacity, DS-hiPSCs were exposed to Accutase at room temperature for 8–10 min and pipetted up and down to ensure that the remaining aggregates were broken up into single cells. Single-cell suspensions were plated in mTeSR^TM^1 with 10 μM Y-27632 at the appropriate density and incubated for 24 hours. For endoderm commitment, DS-hiPSCs were induced by a STEMdiff™ Definitive Endoderm Kit (StemCell Technologies) following the manufacturer's instructions. For mesoderm differentiation, cells were cultured in E8 medium (Cellapy Biotechnology, Beijing, China) with 10 μM CHIR99021 (Tocris Bioscience, St Louis, MO, USA) for 4 days with daily refreshing of the medium. For ectoderm differentiation, cells were induced to differentiate by dual-SMAD inhibition in monolayer culture as described previously [2]. The expression of ectodermal, mesodermal, or endodermal markers of differentiated cells was detected by immunofluorescence staining.

For verification of the *in vivo* pluripotency of DS-hiPSCs, cells were disaggregated with Accutase, and 1 x10^6^ cells were suspended in cold Matrigel. The mixtures including cells and Matrigel were then subcutaneously injected into immunodeficient mice (NOG mice). Tumors that grew for 8–10 weeks were isolated, dissected, fixed in 4% PFA and examined histologically after hematoxylin and eosin staining. All experimental procedures involving animals were approved by the Animal Ethics Committee of Sun Yat-sen University (Approval NO. SYSU-IACUC-2018-000034).

For karyotype analysis, undifferentiated DS-hiPSCs at passage 10 were treated with 50 ng/ml colchicine for 3 hours and then collected. Metaphase chromosomes were investigated by G banding with at least 20 metaphases analyzed for each sample by DaAn Gene Co., Ltd., of Sun Yat-sen University from Guangzhou.

**Neural crest differentiation of hPSCs**

Neural crest differentiation was performed using a monolayer culture method or EB formation-based protocol. For the monolayer differentiation method, confluent hPSCs were dissociated into single cells with Accutase at room temperature for 8–10 min and then replated at a density of 1×10^4^ cells/cm^2^ on Matrigel-coated dishes in completely defined medium (CDM) containing DMEM-F12 as the basal medium and supplemented with 1% N2, 2% B27, 1 mM L-glutamine, 0.1 mM 2-mercaptoethanol, 1% MEN Non-Essential Amino Acid Solution (all from Thermo Fisher Scientific), 20 ng/ml basic fibroblast growth factor (bFGF; PeproTech, Rocky Hill, New Jersey, USA) and 10 μM Y-27632 (Sigma-Aldrich, St. Louis, MO, USA). Twenty-four hours later, the medium was replaced by NCN2, which included DMEM-F12, 1% N2, 1.0 μM CHIR99021, and 0.5 μM SB431542 (both sourced from Tocris Bioscience). The cells were cultured in NCN2 medium for 7 days (D0-D7), and the medium was replaced daily. NCSCs (p75^high^HNK1^+^ cells) were isolated by FACS using a BD Influx Cell Sorter (BD-Pharmingen, Palo Alto, CA, USA), cultured and expanded in NCCM containing 1% N2, 2% B27, 20 ng/ml bFGF, and 20 ng/ml epidermal growth factor (EGF; PeproTech).

For EB formation, an easy, standardized approach using AggreWell^TM^ 800 plates for the generation of EB was adopted (StemCell Technologies). The plates were pretreated according to the manufacturer’s instructions. For neural crest differentiation, undifferentiated hPSC colonies were disassembled into single-cell suspensions by incubation with Accutase. Then, the cells were seeded in the prepared AggreWell^TM^ plate at 1×10^6^ cells per well, and 2 ml of complete medium was added to each well. Finally, the AggreWell^TM^ plate was centrifuged at 100 × g for 3 min to capture cells in the microwells immediately and incubated at 37°C with 5% CO_2_ and 95% humidity. Half of the medium was replaced with fresh complete medium 24 hours later. After 40 hours of incubation, the EBs were harvested from AggreWell^TM^ plates and cultured in ultralow attachment dishes in NCCM for 3 days. Then, the EBs were replated on dishes coated with Matrigel in the same medium for the next 2 days. NCSCs were labeled with antibodies against p75 and HNK1 and collected by FACS using a BD Influx cell sorter.

**Multilineage differentiation of NCSCs**

For peripheral neuron differentiation, NCSCs were plated on Matrigel-coated plates and cultured in neuronal induction medium containing N2, B27, 10 ng/ml brain-derived neurotrophic factor (BDNF; PeproTech), 10 ng/ml glial cell line-derived neurotrophic factor (GDNF; PeproTech), 10 ng/ml nerve growth factor (NGF; PeproTech), 10 ng/ml neurotrophin-3 (NT3; PeproTech), 200 μM L-ascorbic acid (AA; Sigma-Aldrich) and 0.5 mM dibutyryl-cAMP (db-cAMP; Sigma-Aldrich). NCSCs were differentiated for 3–4 weeks and then verified by immunofluorescence staining using anti- PRPH and anti-β-III Tubulin (TUBB3) antibodies (R&D Systems, Minneapolis, MN, USA).

For Schwann cell differentiation, NCSCs were cultured in serum-free medium with 10 ng/ml ciliary neurotrophic factor (CNTF; PeproTech), 20 ng/ml neuregulin (Sigma-Aldrich), and 0.5 mM db-cAMP for 2–3 weeks. The expression of Schwann cell markers was detected by immunofluorescence staining using anti-S100B and anti-GFAP antibodies (Millipore, Temecula, CA, USA),

For MSC differentiation, NCSCs were cultured in MesenCult^TM^-ACF Plus Medium (StemCell Technologies). Three to four weeks later, the resulting cells (NCSC-MSCs) were assessed for the expression of MSC surface markers by FACS and their differentiation abilities to osteoblasts, adipocytes and chondrocytes.

For osteogenic differentiation, NCSC-MSCs were cultured in low glucose-DMEM (L-DMEM) with 10 mM β-glycerol phosphate (β-GP; Sigma-Aldrich), 10 nM dexamethasone (Dex; Sigma-Aldrich) and 200 mM AA (Sigma-Aldrich). Three weeks later, osteogenic differentiation was evaluated by Alizarin Red S staining. For adipogenic differentiation, NCSC-MSCs were cultured in high glucose-DMEM (H-DMEM) with 1 mM Dex (Sigma-Aldrich), 10 μg/ml insulin, and 0.5 mM 3-isobutyl-1-methylxanthine (IBMX; Sigma-Aldrich) for 21–28 days. Adipocyte generation was validated by Oil Red O staining. For chondrogenic differentiation, NCSC-MSCs were cultured in suspension in the bottom of a 15 ml conic tube with a 1-2 ml MesenCult^TM^-ACF Chondrogenic Differentiation Kit (StemCell Technologies) for 3 weeks. The resulting chondrogenic pellets were detected by toluidine blue staining.

**Construction of the lentivectors for RNA silencing or gene overexpression**

In this study, shRNA technology was used for gene knockdown in DS group. Two to three shRNA sequences targeting human CXADR, COL18A1, or SUMO3 followed the guidelines in https://www.invivogen.com/review-sirna-shrna-design were designed, and then introduced into linearized lentiviral vector pLL3.7 separately. Lentivirus was packaged through transfection of 293FT cells with each recombinant pLL3.7 vector (containing GFP-coding sequence) and used for the transduction of DS-hiPSCs. Single GFP positive cells were then isolated by Fluorescence-activated Cell Sorting (FACS) and expanded in vitro. After 2-3 passages, the mRNA and protein expression levels of CXADR was detected. Single cell-derived colonies with successfully CXADR knockdown were selected for further study. An insert-free vector was used as a negative control (designated “shCtrl”). The sequence information of short-hairpin RNAs is listed in Supplementary Table 1.

Lentiviral vectors for the overexpression of CXADR were constructed by GeneCopoeia^TM^ (Guangzhou, China). An insert-free vector was used as a negative control (designated “Ctrl”).

**Lentivirus packaging and transduction of hPSCs**

The lentiviral particles were prepared by transient cotransfection of 293FT cells with the lentiviral vector mentioned above and ViraPower Lentiviral packaging mix (Invitrogen) using Lipofectamine 2000 transfection reagent (Invitrogen) and incubated for 3 days. The supernatant was filtered and subsequently concentrated by ultracentrifugation (50,000 g, for 90 min at 4°C).

For lentivirus infection, hPSCs were dissociated and replated on Matrigel-coated dishes and cultured in mTeSR medium with 10 μM Y-27632 and 5 µg/ml polybrene for approximately 14 hours. Five days after transduction, transduced hPSCs were either purified by GFP expression through FACS or exposed to medium containing 1–5 µg/ml puromycin (Sigma) for another 5 days. The corresponding empty vector was transduced separately into each cell line as a control.

**CCK-8 assay**

For the cell proliferation assay, differentiated cells on day 7 were dissociated by Accutase. Then, 1× 10^4^ cells/cm^2^ were replated on a 96-well plate and cultured for 7 days on the basis of NCSCs induction protocol which was introduced in Fig.1a. Then, CCK-8 reagent (Dojindo, Kumamoto, Japan) was diluted with NCN2 medium in a ratio of 1:10 and added to each well for a 2 hour incubation at 37 °C. The absorbance was measured at 450 nm using an Infinite 200 PRO multimode plate reader (Tecan, Switzerland).

**Immunofluorescence staining**

Cells were fixed with 4% paraformaldehyde for 15 min before permeabilization with 0.3% Triton X-100 (Sigma) for 30 min. Cells were incubated with primary and secondary antibodies (diluted with the same buffer containing 10% goat serum or 1% BSA in TBS) at 4°C overnight and at room temperature for 2 hours, respectively. The nuclei were stained with 4',6-diamidino-2-phenylindole (DAPI; Sigma-Aldrich) for 15 min before mounting. Images were observed and analyzed using confocal microscopy. The primary and secondary antibodies used in immunostaining are listed in Supplementary Table 2.

**qPCR**

Total RNA was extracted from FACS-sorted cells using TRIzol (Invitrogen), and cDNA was generated using Multiscribe reverse transcriptase (Invitrogen). qPCR was carried out using 20 ng cDNA in a 20-µl reaction system with a LightCycler 480 Detection System (Roche Diagnostics, Branchburg, NJ, USA). The thermocycling conditions consisted of predenaturation at 95°C for 5 min, followed by 40 cycles of denaturation at 95°C for 15 s, annealing at 60°C for 30 s, and extension at 72°C for 30 s. GAPDH was amplified as an internal control, and gene expression levels were calculated as fold changes. Primer sequences are listed in Supplementary Table 3.

**Western blot**

For protein extraction, cells were washed with cold PBS and lysed in 1x RIPA buffer containing phenylmethanesulfonyl fluoride (PMSF) on ice for 30 min. The lysate was collected with a cell scraper, sonicated, and then centrifuged at 16,000 × g for 10 min at 4°C. Equal amounts of proteins were separated by SDS-PAGE and electrotransferred to 0.45-μm pore-sized polyvinylidene difluoride membranes (Millipore, Bedford, MA, USA). Then, each membrane was blocked using 3% BSA solution of 0.1% (v/v) Tween-20/TBS (TBS/T) for 1 hour at room temperature and then incubated with appropriate primary antibodies diluted with TBS/T containing 1% BSA overnight at 4°C, followed by reaction with horseradish peroxidase (HRP)-linked secondary antibodies (Cell Signaling Technology, Beverly, MA, USA) at room temperature for 1 hour before chemiluminescence detection. Information on the antibodies used in western blotting is listed in Supplementary Table 4.

**Flow cytometry**

Differentiated cells were detached with Accutase, suspended in cold PBS buffer containing 0.1% bovine serum albumin (BSA), and then incubated with monoclonal antibodies (all from BD Biosciences) against human antigens, including p75, HNK1, CD73, CD105, CD166, CD29, CD44, CD34, or CD45. Isotype controls were adopted as negative controls. Flow cytometric results were analyzed using CytExpert (Beckman Coulter) or FlowJo software (BD Biosciences). Information on the antibodies used in FACS is listed in Supplementary Table 5.

**RNA sequencing**

Total RNA was extracted from control NCSCs, DS-NCSCs, and shCXADR DS-NCSCs using TRIzol reagent (Invitrogen, Carlsbad, CA) according to the manufacturer’s instructions. The RNA libraries were prepared from the total RNA and constructed using an Illumina mRNA-seq Prep Kit (Illumina, San Diego, CA, USA). The fragmented and randomly primed 150 bp paired-end libraries were sequenced using Illumina HiSeq 2000. Sequencing data were analyzed using Consensus Assessment of Sequence and Variation (CASAVA, version 1.8.2; Illumina) and IPA software (Ingenuity Systems, Inc., Redwood City, CA, USA) to categorize the differentially regulated genes. In brief, all differentially expressed genes (DEGs; with RPKM value more than 1) were identified using fold-change cutoffs (default = 1.5), p-value cutoffs (calculated using ANOVA or linear regression, with ANOVA p-value < 0.05 as the default setting). Genes located on HSA21, which were expressed (RPKM value more than 1) in one sample at least, were enrolled in the analysis. Fold changes of these genes between DS cells and control cells were calculated respectively.

**Generation of CXADR-knockout hiPSCs**

Single-guide RNAs (SgRNAs) targeting the human CXADR locus were designed using E-CRISP tools and cloned into the CRISPR-Cas9 expression vector pSpCas9 (BB)-2A-GFP (PX458). The editing efficiency was evaluated in 293FT cells and the most efficient SgRNA (SgRNA1) was selected for the transfection of DS-hiPSCs. Single cell-derived colonies with significant down regulation of CXADR expression were chosen for functional analysis. The sequence information of Single-guide RNAs is listed in Supplementary Table 6.

**Statistical analysis**

All experiments were replicated at least 3 times, and each experiment was performed in triplicate. Data are represented as the mean ± s.e.m. Student’s t test was applied when two independent groups were compared, while one-way analysis of variance (ANOVA) was employed to compare three or more groups. A P value < 0.05 was considered to be statistically significant.

**References**

1. Li W, Huang L, Zeng J, Lin W, Li K, Sun J *et al.*. Characterization and transplantation of enteric neural crest cells from human induced pluripotent stem cells. *Mol Psychiatry* 2018 2018-03-01; **23**(3)**:** 499-508.

2. Chambers SM, Fasano CA, Papapetrou EP, Tomishima M, Sadelain M, Studer L. Highly efficient neural conversion of human ES and iPS cells by dual inhibition of SMAD signaling. *NAT BIOTECHNOL* 2009 2009-03-01; **27**(3)**:** 275-280.
