# Supplementary material for: Migration deficits of the neural crest caused by CXADR triplication in a human Down syndrome stem cell model
Source: Cell Death Dis. 2022 Dec 5;13(12):1018. doi: 10.1038/s41419-022-05481-6 (PMC9722909; doi:10.1038/s41419-022-05481-6)
Supplement: Supplementary file 18 — Supplementary table 1 [file 41419_2022_5481_MOESM18_ESM.docx]

**Supplementary Table 1. Sequence information of Short-hairpin RNAs**

| **Gene** | **Direction** | **Sequences** |
| --- | --- | --- |
| *COL18A1-1* | Forward | 5’ TGC CTC TTC TTC CGT GAC TTC TCT TCC TGT CAA GAA GTC ACG GAA GAA GAG GCT TTT TTC 3’ |
|  | Reverse | 5’ TCG AGA AAA AAG CCT CTT CTT CCG TGA CTT CTT GAC AGG AAG AGA AGT CAC GGA AGA AGA GGC A 3’ |
| *COL18A1-2* | Forward | 5’ TAC CTG AAC TGG CTT TGG TTC ACT TCC TGT CAT GAA CCA AAG CCA GTT CAG GTT TTT TTC 3’ |
|  | Reverse | 5’ TCG AGA AAA AAA CCT GAA CTG GCT TTG GTT CAT GAC AGG AAG TGA ACC AAA GCC AGT TCA GGT A 3’ |
| *COL18A1-3* | Forward | 5’ TGC CTC TTC TTC CGT GAC TTC TCT TCC TGT CAA GAA GTC ACG GAA GAA GAG GCT TTT TTC 3’ |
|  | Reverse | 5’ TCG AGA AAA AAG CCT CTT CTT CCG TGA CTT CTT GAC AGG AAG AGA AGT CAC GGA AGA AGA GGC A 3’ |
| *CXADR-1* | Forward | 5’ TGA AGC TAC ATC GGC AGT AAT CTT CCT GTC AAT TAC TGC CGA TGT AGC TTC TTT TTT C 3’ |
|  | Reverse | 5’ TCG AGA AAA AAG AAG CTA CAT CGG CAG TAA TTG ACA GGA AGA TTA CTG CCG ATG TAG CTT CA 3’ |
| *CXADR-2* | Forward | 5’ TGA AGC TAC ATC GGC AGT AAT CTT CCT GTC AAT TAC TGC CGA TGT AGC TTC TTT TTT C 3’ |
|  | Reverse | 5’ TCG AGA AAA AAG AAG CTA CAT CGG CAG TAA TTG ACA GGA AGA TTA CTG CCG ATG TAG CT TCA 3’ |
| *SUMO3-1* | Forward | 5’ TGT CAA TGA GGC AGA TCA GAT CTT CCT GTC AAT CTG ATC TGC CTC ATT GAC TTT TTT C 3’ |
|  | Reverse | 5’ TCG AGA AAA AAG TCA ATG AGG CAG ATC AGA TGA CAG GAA GAT CTG ATC TGC CTC ATT GAC A 3’ |
| *SUMO3-2* | Forward | 5’ TGA CGG GCA GCC AAT CAA TGA CTT CCT GTC ATC ATT GAT TGG CTG CCC GTC TTT TTT C 3’ |
|  | Reverse | 5’ TCG AGA AAA AAG TCA ATG AGG CAG ATC AGA TGA CAG GAA GAT CTG ATC TGC CTC ATT GAC A 3’ |
