# Supplementary material for: Migration deficits of the neural crest caused by CXADR triplication in a human Down syndrome stem cell model
Source: Cell Death Dis. 2022 Dec 5;13(12):1018. doi: 10.1038/s41419-022-05481-6 (PMC9722909; doi:10.1038/s41419-022-05481-6)
Supplement: Supplementary file 19 — Supplementary table 2 [file 41419_2022_5481_MOESM19_ESM.docx]

**Supplementary Table 2. Antibodies used in Immunofluorescence Staining**

| **Antigen** | **Host** | **Dilution** | **Company** | **Cat. No.** |
| --- | --- | --- | --- | --- |
| Active caspase3 | rabbit | 1:20 | abcam | ab2302 |
| BRACHYURY(TBXT) | goat | 1:200 | R&D System | AF2085 |
| GFAP | rabbit | 1:500 | Millipore | ab5804 |
| HNK1 | mouse | 1:200 | Sigma-Aldrich | c6680 |
| Ki67 | rabbit | 1:1000 | abcam | ab15580 |
| NANOG | rabbit | 1:200 | Cell signaling technology | 3580 |
| OCT4 | rabbit | 1:200 | Santa cruz | Sc-9081 |
| SSEA4 | mouse | 1:200 | EMD Millipore | MAB4304 |
| SOX17 | goat | 1:100 | R&D System | AF1924 |
| SOX10 | rabbit | 1:200 | abcam | ab155279 |
| SOX10 | goat | 1:100 | R&D System | AF2864 |
| S100B | mouse | 1:500 | Novus Biologicals | SA-12 |
| peripherin | rabbit | 1:200 | abcam | ab123576 |
| p75 | rabbit | 1:200 | Promega | G3231 |
| TUNEL | - | - | Roche | 11684795910 |
| TUBB3 | mouse | 1:500 | R&D System | MAB1195 |
| Goat anti-mouse IgG Alexa 488 | goat | 1:1000 | Invitrogen | A11001 |
| Goat anti-rabbit IgG Alexa 488 | goat | 1:1000 | Invitrogen | A11008 |
| Goat anti-rabbit IgG Alexa 555 | goat | 1:1000 | Invitrogen | A21428 |
| Goat anti-mouse IgG Alexa 555 | goat | 1:1000 | Invitrogen | A21422 |
| Donkey anti-goat IgG Alexa 488 | donkey | 1:1000 | Invitrogen | A32814 |
