# Supplementary material for: Migration deficits of the neural crest caused by CXADR triplication in a human Down syndrome stem cell model
Source: Cell Death Dis. 2022 Dec 5;13(12):1018. doi: 10.1038/s41419-022-05481-6 (PMC9722909; doi:10.1038/s41419-022-05481-6)
Supplement: Supplementary file 20 — Supplementary table 3 [file 41419_2022_5481_MOESM20_ESM.docx]

**Supplementary Table 3. Primers used for qRT-PCR**

| **Gene** | **Direction** | **Sequences** |
| --- | --- | --- |
| *CDH1* | Forward | 5’ ATT TTT CCC TCG ACA CCC GAT 3’ |
|  | Reverse | 5’ TCC CAG GCG TAG ACC AAG A 3’ |
| *CLDN7* | Forward | 5’ AGC TGC AAA ATG TAC GAC TCG 3’ |
|  | Reverse | 5’ GGA GAC CAC CAT TAG GGC TC 3’ |
| *COL18A1* | Forward | 5’ CAG TGG ACA CAC TTA GCC CTC 3’ |
|  | Reverse | 5’ GCG GCA TTC TCT GGA ACT CC 3’ |
| *CXADR* | Forward | 5’ GTG CTC CTG TGC GGA GTA G 3’ |
|  | Reverse | 5’ ATG GCA GAT AGG CAG TTT CCC 3’ |
| *DLX5* | Forward | 5’ TTC CAA GCT CCG TTC CAG AC 3’ |
|  | Reverse | 5’ GAA TCG GTA GCT GAA GAC TCG 3’ |
| *DSCAM* | Forward | 5’ TTT TAC GGG AGC CCT ATA CAG T 3’ |
|  | Reverse | 5’ TGC ACT TGA AGA CCG CAA CAT 3’ |
| *ETS1* | Forward | 5’ TAC ACA GGC AGT GGA CCA ATC 3’ |
|  | Reverse | 5’ CCC CGC TGT CTT GTG GAT G 3’ |
| *GAPDH* | Forward | 5’ GAA GGT GAA GGT CGG AGT C 3’ |
|  | Reverse | 5’ GAA GAT GGT GAT GGG ATT TC 3’ |
| *HOXA1* | Forward | 5’ TCC TGG AAT ACC CCA TAC TTA GC 3’ |
|  | Reverse | 5’ GCA CGA CTG GAA AGT TGT AAT CC 3’ |
| *LHX5* | Forward | 5’ GCG CGT GGC ACA TCA AAT G 3’ |
|  | Reverse | 5’ GCC AAA GCG CCT GAA AAA GTC 3’ |
| *PAK1* | Forward | 5’ CAG CCC CTC CGA TGA GAA ATA 3’ |
|  | Reverse | 5’ CAA AAC CGA CAT GAA TTG TGT GT 3’ |
| *p75* | Forward | 5’ CTG CCT GGA CAG CGT GAC GTT 3’ |
|  | Reverse | 5’ GCA GCG CCC AGT CGT CTC AT 3’ |
| *SOX10* | Forward | 5’ CCC GCA CTA CAC CGA CCA 3’ |
|  | Reverse | 5’ AGG AGA AAG CCG AGT AGA 3’ |
| *SOX9* | Forward | 5’ AGC GAA CGC ACA TCA AGA C 3’ |
|  | Reverse | 5’ CTG TAG GCG ATC TGT TGG GG 3’ |
| *SNAI2* | Forward | 5’ CGA ACT GGA CAC ACA TAC AGT G 3’ |
|  | Reverse | 5’ CTG AGG ATC TCT GGT TGT GGT 3’ |
| *SUMO3* | Forward | 5’ GAATGACCACATCAACCTGAAGG 3’ |
|  | Reverse | 5’ GCCCGTCGAACCTGAATCT 3’ |
