# Supplementary material for: Migration deficits of the neural crest caused by CXADR triplication in a human Down syndrome stem cell model
Source: Cell Death Dis. 2022 Dec 5;13(12):1018. doi: 10.1038/s41419-022-05481-6 (PMC9722909; doi:10.1038/s41419-022-05481-6)
Supplement: Supplementary file 21 — Supplementary table 4 [file 41419_2022_5481_MOESM21_ESM.docx]

**Supplementary Table 4. Antibodies used in Western blotting**

| **Antigen** | **Host** | **Company** | **Cat. No.** |
| --- | --- | --- | --- |
| CXADR | rabbit | Cell Signaling Technology | 16984 |
| COL18A1 | rabbit | abcam | ab207162 |
| ECAD | mouse | ThermoFisher scientific | 13-5700 |
| GAPDH | rabbit | Cell Signaling Technology | 2118s |
| SUMO3 | rabbit | abcam | ab34661 |
| anti-mouse IgG HRP-linked Ab | goat | Cell Signaling Technology | 7056 |
| anti-rabbit IgG HRP-linked Ab | goat | Cell Signaling Technology | 7074 |
