# Supplementary material for: Migration deficits of the neural crest caused by CXADR triplication in a human Down syndrome stem cell model
Source: Cell Death Dis. 2022 Dec 5;13(12):1018. doi: 10.1038/s41419-022-05481-6 (PMC9722909; doi:10.1038/s41419-022-05481-6)
Supplement: Supplementary file 22 — Supplementary table 5 [file 41419_2022_5481_MOESM22_ESM.docx]

**Supplementary Table 5. Antibodies used in FACS**

| **Antigen** | **Label** | **Company** | **Cat. No.** |
| --- | --- | --- | --- |
| Anti-human CD271 (p75) | AF647 | BD Pharmingen | 560326 |
| Anti-human CD57 (HNK1) | PE | BD Pharmingen | 560844 |
| Anti-human CD57 (HNK1) | PE-CY7 | eBioscience | 25-0577-42 |
| Anti-human CD73 | PE-CY7 | BD Pharmingen | 561285 |
| Anti-human CD105 | FITC | BD Pharmingen | 561443 |
| Anti-human CD166 | FITC | Invitrogen | MA5-23565 |
| Anti-human CD29 | PE | BD Pharmingen | 556049 |
| Anti-human CD44 | APC | BD Pharmingen | 559942 |
| Anti-human CD34 | FITC | eBioscience | 11-0349-42 |
| Anti-human CD45 | PE | BD Pharmingen | 555483 |
