# Supplementary material for: Migration deficits of the neural crest caused by CXADR triplication in a human Down syndrome stem cell model
Source: Cell Death Dis. 2022 Dec 5;13(12):1018. doi: 10.1038/s41419-022-05481-6 (PMC9722909; doi:10.1038/s41419-022-05481-6)
Supplement: Supplementary file 23 — Supplementary table 6 [file 41419_2022_5481_MOESM23_ESM.docx]

**Supplementary Table 6. Sequence information of Single-guide RNAs for targeting human CXADR**

| **Name** | **Sequences** |
| --- | --- |
| *SgRNA1* | 5’ TCC AGC GGT CCC TGG TCT T 3’ |
| *SgRNA2* | 5’ GAC TAA GCG TAA ATT TGC A 3’ |
